# Characteristics of and meningococcal disease prevention strategies for commercially insured persons receiving eculizumab in the United States

**DOI:** 10.1371/journal.pone.0241989

**Published:** 2020-11-12

**Authors:** Catherine H. Bozio, Cheryl Isenhour, Lucy A. McNamara

**Affiliations:** 1 Division of Bacterial Diseases, National Center for Immunization and Respiratory Diseases, CDC, Atlanta, GA, United States of America; 2 Division of Health Informatics and Surveillance, Center for Surveillance, Epidemiology, and Laboratory Services, CDC, Atlanta, GA, United States of America; Universidad Nacional de la Plata, ARGENTINA

## Abstract

**Introduction:**

Eculizumab is a licensed treatment for several rare, complement-mediated diseases. Eculizumab use is associated with an approximately 2,000-fold increased meningococcal disease risk. In the United States, meningococcal vaccines are recommended for eculizumab recipients but there are no recommendations on use of long-term antibiotic prophylaxis. We describe characteristics of and meningococcal vaccine and antibiotic receipt in U.S. eculizumab recipients to inform meningococcal disease prevention strategies.

**Methods:**

Persons in the IBM® MarketScan® Research Databases with ≥1 claim for eculizumab injection during 2007–2017 were included. Indication for eculizumab use, meningococcal vaccine receipt, and antibiotic receipt were assessed using International Classification of Diseases-9/10 diagnosis codes, vaccine administration procedure codes, and antibiotic codes from pharmacy claims, respectively.

**Results:**

Overall 696 persons met the inclusion criteria. Paroxysmal nocturnal hemoglobinuria (PNH) and atypical hemolytic uremic syndrome (aHUS) were the most common indications for eculizumab use (41% and 37%, respectively); 20% had an undetermined indication. From June 2015 through December 2017, 28% (41/148) of continuously-enrolled patients received ≥1 serogroup B vaccine dose. For serogroup ACWY conjugate vaccine, 45% (91/201) of patients received ≥1 dose within five years of their most recent eculizumab dose, as recommended. Of eculizumab recipients with outpatient prescription data, 7% (41/579) received antibiotics for ≥50% of the period of increased risk for meningococcal disease.

**Conclusion:**

Many eculizumab recipients had an undetermined indication for eculizumab use; few were up-to-date for recommended meningococcal vaccines or were prescribed antibiotics long-term. These findings can inform further investigation of how to best protect this population from meningococcal disease.

## Introduction

Eculizumab, a terminal complement inhibitor, is licensed in the United States for treatment of four rare, life-threatening illnesses: paroxysmal nocturnal hemoglobinuria (PNH) (since 2007), atypical hemolytic uremic syndrome (aHUS) (2011), generalized myasthenia gravis (gMG) (2017), and neuromyelitis optica spectrum disorder (2019). These illnesses have few other treatments, and long-term treatment may be expected for many persons receiving eculizumab.

The complement pathway is critical for defense against invasive disease caused by the bacterium *Neisseria meningitidis*, and inherited deficiencies in the terminal complement pathway (C5 to C9) are associated with an up to 10,000-fold increase incidence of meningococcal disease. By blocking complement component C5, eculizumab similarly inhibits serum bactericidal activity against *Neisseria meningitidis*. The U.S. prescribing information for eculizumab approved by the Food and Drug Administration includes a boxed warning for increased risk of meningococcal disease in recipients [1]. Disseminated gonococcal infections and invasive infections due to other *Neisseria* species have also been reported in eculizumab recipients [2, 3].

In the United States, the Advisory Committee on Immunization Practices (ACIP) has recommended the quadrivalent meningococcal conjugate (MenACWY) vaccine for persons with complement deficiency, including those receiving eculizumab or other complement inhibitors, since 2005 [4]. Serogroup B vaccines have also been recommended for this population since June, 2015 [5]. However, there have been numerous reports of eculizumab recipients developing meningococcal disease caused by serogroups that they have been vaccinated against, as well as disease caused by nongroupable meningococcal strains which may not be covered by available vaccines [6–9]. These findings demonstrate that meningococcal vaccines do not adequately protect these high-risk individuals from meningococcal disease.

In addition to vaccination, long-term antibiotic prophylaxis for eculizumab recipients is recommended in the United Kingdom [10] and France [11]. Although the CDC has advised that prescribers could consider using antibiotic prophylaxis in eculizumab recipients for the duration of eculizumab therapy [6], there are no official recommendations on this practice in the United States and no data are available on the effectiveness of long-term antibiotic prophylaxis among eculizumab recipients. Whether and how frequently providers may prescribe long-term antibiotics for eculizumab recipients in the United States are unknown. To inform potential guidance on long-term antibiotic prophylaxis for eculizumab recipients in the United States and to assess the feasibility of potential studies of prophylaxis effectiveness, additional information is needed about the characteristics of the patients receiving eculizumab and their meningococcal vaccine and antibiotic use. We used commercial insurance claims data to describe eculizumab recipients in the United States to inform meningococcal disease prevention strategies for this population.

## Methods

### Cohort definition

**Fig 1:**
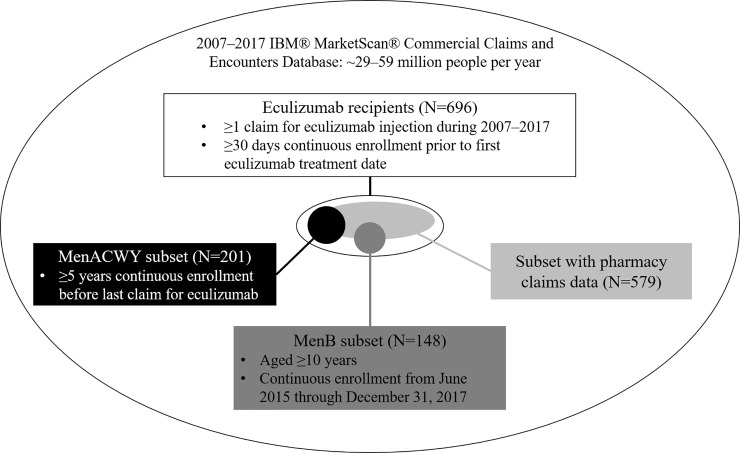
Inclusion criteria for full analytic cohort and sub-cohorts in the IBM Marketscan Commercial Database, 2007–2017.

For our analysis we used the 2007–2017 IBM MarketScan Commercial Database, which includes claims for approximately 25% of U.S. persons who have employer-sponsored insurance. Eculizumab recipients were identified as individuals with ≥1 claim with Healthcare Common Procedure Coding System or National Drug Codes for eculizumab (S1 Table) and continuous enrollment of ≥30 days prior to first eculizumab claim (Fig 1).

Eculizumab recipients were initially screened for the licensed indications for eculizumab as of 2017, PNH, aHUS, and gMG, using International Classification of Diseases (ICD) 9/10 diagnosis codes (S2 Table) on claims during the 30 days before to 14 days after the first eculizumab claim (hereafter referred to as the defined six-week window). Eculizumab recipients were then classified into indication categories for eculizumab use: PNH, aHUS, gMG, multiple indications (a combination of PNH, aHUS, or gMG), or undetermined. Individuals with aHUS codes were further screened for typical HUS using additional ICD codes (S2 Table), and those with typical HUS were reclassified as such. Individuals with undetermined indication were screened again for PNH, aHUS, and gMG codes on claims at any point during enrollment and then reclassified into an indication category. For individuals who had codes for multiple indications, persons were reclassified as having one of the two conditions if they had ≥10 times more claims for one condition than the other. All remaining individuals with no codes for aHUS, PNH, or gMG at any point during enrollment were classified as having an undetermined indication for eculizumab use.

### Defining medical conditions, vaccine receipt, and antibiotic use

We looked for ICD-9/10 codes for specific conditions among eculizumab recipients based on conditions that are associated with licensed indications [12], indications for eculizumab use previously reported to CDC (CDC unpublished data), and the most common conditions found on eculizumab claims in our cohort. Specific co-morbidities among patients around the time of beginning eculizumab treatment were identified based on presence of ≥1 ICD-9/10 code (S2 Table) on any claim in the defined six-week window of the first eculizumab injection. The six-week window was used to balance sensitivity to detect conditions at the time that patients began eculizumab treatment with avoiding identifying many conditions that resolved or began far from the time that patients began using eculizumab. We defined an individual as being diagnosed with meningococcal disease, gonococcal disease, meningitis not otherwise specified (NOS), and sepsis NOS based on the presence of ≥1 ICD-9/10 code (S2 Table) on any claim between first documented eculizumab treatment and 90 days after the last eculizumab injection (hereafter referred to as the meningococcal disease risk period) [1] or end of enrollment, whichever was shorter. For persons with codes for meningitis and/or sepsis NOS, we examined all codes on claims for that hospitalization to identify potential etiologies.

Eculizumab recipients are recommended to receive MenACWY booster doses every 3–5 years depending on age [13], and a MenB primary series has been recommended for eculizumab recipients aged ≥10 years since June, 2015 [5]. Separate analytic sub-cohorts were created with additional requirements (Fig 1) to examine MenACWY (including polysaccharide and conjugate vaccines) and MenB vaccine receipt using vaccine codes (S1 Table).

Outpatient antibiotic use was examined among eculizumab recipients with a pharmacy claims feed (Fig 1). We identified outpatient prescriptions for penicillin, ciprofloxacin, or macrolide antibiotics (S1 Table) during an individual’s meningococcal disease risk period. These three antibiotics were assessed based on (1) recommendations in other countries to use penicillin or macrolides for long-term prophylaxis among eculizumab recipients [10] and (2) common use of ciprofloxacin for short-term meningococcal disease prophylaxis, which is recommended in the 2019 US prescribing information for Soliris® for individuals starting eculizumab therapy prior to receiving meningococcal vaccination [1].

### Statistical analysis

Differences in characteristics across indications for eculizumab use were compared using the chi-square test. Differences in the median enrollment times across indications were compared using the Kruskal-Wallis test. Median therapy duration was estimated overall and by indication using Kaplan-Meier survival curves. For persons who had <1 month of enrollment after the last documented eculizumab injection, treatment duration was censored at the end of the enrollment period. All analyses were performed in SAS 9.4.

This secondary analysis of deidentified insurance claims data did not require Institutional Review Board approval.

## Results

### Characteristics of eculizumab recipients

Between January 1, 2007 and December 31, 2017, 696 persons met our inclusion criteria (Fig 1). PNH and aHUS were the most common indications for eculizumab use (41.0% and 37.1%, respectively); 139 (20.0%) persons had an undetermined indication. The number of persons receiving eculizumab for PNH or aHUS increased over the first 3–5 years following licensure for these indications, then plateaued; meanwhile the number of persons receiving eculizumab for an undetermined indication remained constant (S1 Fig). Four (0.6%) persons had gMG, three (0.4%) had typical HUS, and seven (1.0%) had multiple indications. Given the small numbers in these latter categories, only those with PNH, aHUS, and undetermined indications were included in subsequent comparisons.

The median patient age at first documented use of eculizumab was 41 years (range: 0–88), and 59.8% were female. Among 87 patients aged <18 years, 14 were aged <5 years, including four infants aged <1 year; one infant had aHUS and three had an undetermined indication for eculizumab use. Another 15 patients were aged 5–<10 years and 58 were aged 10–<18 years. The age and sex distributions significantly varied by indication for eculizumab use (Table 1). The most common insurance plan type was preferred provider organization (56.8%), though plan type differed by indication (p<0.0001; Table 1). The median enrollment time was 1,461 days (interquartile range: 730.5–2557) and was similar across indications (Table 1).

**Table 1:** Demographic characteristics of eculizumab recipients in the IBM Marketscan Commercial Database, overall and by indication for eculizumab use, 2007–2017.

Characteristic	All eculizumab recipients (N = 696)*		Atypical hemolytic uremic syndrome (aHUS) (N = 258)		Paroxysmal nocturnal hemoglobinuria (PNH) (N = 285)		Undetermined indication for eculizumab use (N = 139)		P-value across indications
	N	%	N	%	N	%	N	%	
Age group (years)									
<18	87	12.5	36	14.0	21	7.4	29	20.9	0.0002
18–29	142	20.4	62	24.0	57	20.0	22	15.8	
30–39	100	14.4	40	15.5	48	16.8	11	7.9	
40–49	128	18.4	37	14.3	63	22.1	23	16.6	
50–59	112	16.1	36	14.0	48	16.8	25	18.0	
60–69	85	12.2	37	14.3	32	11.2	14	10.1	
≥70	42	6.0	10	3.9	16	5.6	15	10.8	
Sex									
Male	280	40.2	86	33.3	128	44.9	62	44.6	0.01
Female	416	59.8	172	66.7	157	55.1	77	55.4	
Insurance Plan Type									
Health Management Organization and Point-Of-Service (POS) with capitation	101	14.5	23	8.9	39	13.7	37	26.6	0.00005
Preferred Provider Organization	395	56.8	153	59.3	163	57.2	69	49.6	
Consumer Directed Health Plans and High Deductible Health Plans	79	11.4	39	15.1	33	11.6	6	4.3	
Other**	96	13.8	36	14.0	36	12.6	23	16.6	
Missing	25	3.6	7	2.7	14	4.9	4	2.9	
Total enrollment time (days), median (interquartile range)	1461	(730.5–2557)	1461	(730–2588)	1461	(730–2557)	1461	(823–2192)	0.97

*Among the 696 eculizumab recipients, fourteen are included in only this column of the table because they had ICD codes either for multiple indications (N = 7), for typical HUS (N = 3), or for generalized myasthenia gravis (n = 4).

**Comprehensive, POS without capitation, exclusive provider organization.

Among all eculizumab recipients, the median duration of eculizumab treatment was 711 days (95% confidence interval (CI): 494–1092); however, treatment duration differed substantially by indication (S2 Fig). Persons with PNH had the longest median duration of 2,695 days (95% CI ≥ 1,694; upper limit could not be estimated as 46.5% of patients were still receiving eculizumab at the end of the enrollment period). For persons with aHUS, the median treatment duration was 654 days (95% CI: 467–910). Median duration was 0 days (one infusion) for persons with an undetermined indication; confidence intervals could not be estimated because 68.4% of these patients only received one eculizumab injection.

Aside from the conditions classified as indications for eculizumab, the most common conditions identified within the defined six-week window of first documented eculizumab treatment were kidney conditions (excluding kidney transplant) (29.0%), cancer (25.9%), thrombotic microangiopathy (TMA) (21.0%), and aplastic anemia (20.1%) (Table 2). The distribution of conditions differed by indication: persons with aHUS had a higher proportion of kidney conditions and TMA claims (59.7% and 49.6%, respectively) while aplastic anemia was most common in persons with PNH (37.5%) (Table 2). Persons with aHUS or an undetermined indication had higher frequencies of transplant claims (18.6% and 15.1%, respectively). We found a surprisingly high prevalence of joint-related diagnoses on claims among eculizumab recipients (10.6%), with the highest proportion among persons with an undetermined indication (20.1%) (Table 2).

**Table 2:** Underlying conditions within the defined six-week window of 30 days before to 14 days after first documented eculizumab treatment among eculizumab recipients in the IBM Marketscan Commercial Database, 2007–2017.

Diagnoses	All eculizumab recipients (N = 696)		Atypical hemolytic uremic syndrome (aHUS) (N = 258)		Paroxysmal nocturnal hemoglobinuria (PNH) (N = 285)		Undetermined indication for eculizumab use (N = 139)		P-value from chi-square test across indications
	N	%	N	%	N	%	N	%	
Aplastic anemia	140	20.1	26	10.1	107	37.5	5	3.6	<0.0001
Other hemolytic anemia	34	4.9	17	6.6	16	5.6	0	0.0	0.01
Thrombotic microangiopathy	146	21.0	128	49.6	3	1.1	9	6.5	<0.0001
Other causes of thrombotic microangiopathy*	63	9.1	48	18.6	5	1.8	7	5.0	<0.0001
Any transplant	77	11.1	48	18.6	6	2.1	21	15.1	<0.0001
Kidney	54	7.8	36	14.0	1	0.4	17	12.2	<0.0001
Liver	5	0.7	1	0.4	1	0.4	3	2.7	0.09
Bone marrow	13	1.9	8	3.1	0	0.0	3	2.7	0.01
Other	23	3.3	14	5.4	4	1.4	4	2.9	0.03
Cancer	180	25.9	65	25.2	71	24.9	42	30.2	0.46
Blood	44	6.3	21	8.1	12	4.2	10	7.2	0.15
All other cancers	151	21.7	50	19.4	64	22.5	36	25.9	0.32
Other kidney conditions (excluding kidney transplant)	202	29.0	154	59.7	20	7.0	25	18.0	<0.0001
C3G/membranoproliferative glomerulonephritis	4	0.01	3	1.2	0	0.0	1	0.7	0.20
Neuromyelitis optica	1	0.001	0	0.0	0	0.0	1	0.7	0.14
Joint-related diagnoses	74	10.6	25	9.7	20	7.0	28	20.1	0.0002

*Disseminated intravascular coagulation, severe pre-eclampsia, systemic sclerosis (including progressive and unspecified systemic sclerosis), primary hypercoagulable state, HELLP (hemolysis, elevated liver enzymes and low platelet count), antiphospholipid syndrome, systemic lupus erythematosus.

### Meningococcal vaccine receipt

In the MenACWY sub-cohort, 45.3% (91/201) of patients had claims for ≥1 MenACWY dose within five years of their most recent eculizumab dose (S3 Fig). MenACWY uptake was significantly higher in persons with PNH (58.9%) than in persons with aHUS or an undetermined indication (31.7% and 33.3%, respectively) (p = 0.0008; S3 Fig). From June 2015 through December 2017, 27.7% (41/148) of continuously-enrolled patients aged ≥10 years had claims for ≥1 MenB dose (S3 Fig). Uptake was significantly higher in persons aged 16–23 years (13/25, 56.0%) compared to those aged 10–15 years or ≥24 years (33.3% and 21.1%, respectively) (p = 0.0018). Approximately one-third of persons who started the MenB primary series, or 10.1% (15/148) of eculizumab recipients in the cohort, completed the series. MenB uptake or completion of the MenB primary series did not significantly differ across indications (S3 Fig).

### Antibiotic use during period of increased risk for meningococcal disease

Of the 579 eculizumab recipients with outpatient prescription data, 309 (53.4%) had claims for penicillin (195, 33.7%), ciprofloxacin (135, 23.3%), or macrolide (142, 24.5%) antibiotics at any point during their period of increased risk for meningococcal disease (i.e., date of first eculizumab injection– 3 months after last injection). One hundred ninety-nine persons (34.3%) had claims for antibiotics for <10% of their risk period, 69 (11.9%) for 10–<50% of their risk period, and only 41 (7.1%) for ≥50% of their risk period. Persons who had a transplant claim in the defined six-week window of first documented eculizumab therapy were more likely to have claims for antibiotics for ≥50% of their risk period than those who did not have a transplant claim (17.7% vs. 5.7%, respectively). Persons aged <18 years also more frequently had claims for antibiotics for ≥50% of their risk period relative to those aged ≥18 years (18.2% vs 5.4%, respectively).

**Fig 2:**
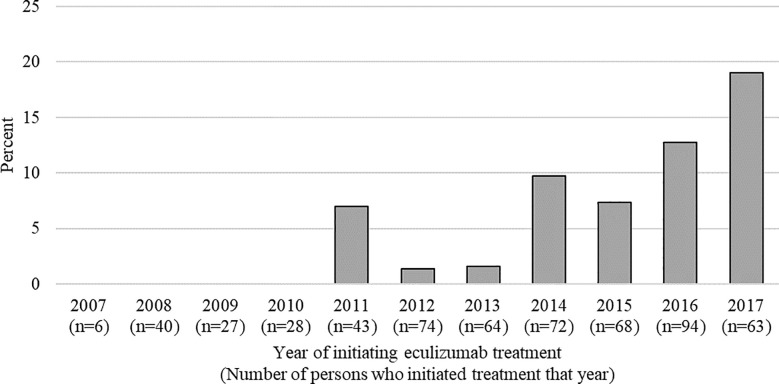
Percentage of eculizumab recipients receiving antibiotics for ≥50% of the risk period by year of starting eculizumab treatment, IBM Marketscan Commercial Database, 2007–2017.

No patients who had first documented eculizumab therapy during 2007–2010 received antibiotics for ≥50% of their risk period. Since then, increases were observed in both the number of persons who had their first documented eculizumab treatment and the percentage receiving antibiotics for ≥50% of their risk period, which increased from 7.0% in 2011 to 19.1% in 2017 (Fig 2). This increasing percentage may be partially due to the increasing proportion of persons with documented eculizumab treatment for <1 year due to censoring at the end of 2017.

### Diagnosis of meningococcal disease, meningitis, and sepsis

One person with aHUS had an inpatient claim for meningococcemia (serogroup unavailable) in 2015, two months after stopping eculizumab treatment (S3 Table). No MenACWY vaccination codes were identified in claims before or after the meningococcemia diagnosis, although as claims were available only for the time period included in Marketscan, it remains possible that this individual received MenACWY vaccination prior to Marketscan enrollment. However, this person did have claims for the complete MenB primary series, which was initiated two months after the claim for meningococcemia. No persons had claims for gonococcal disease during their risk period.

Three persons, one with aHUS and two with PNH, had inpatient claims for meningitis NOS; review of hospitalization claims for these individuals revealed no potential etiologies for the meningitis diagnosis. Forty persons, 19 with PNH, 18 with aHUS, and 3 with undetermined indication for eculizumab use, had diagnosis codes for sepsis NOS; six persons had information on potential etiology noted in inpatient claims, including two with gram-negative bacteria with no specific pathogen indicated (S3 Table). Four persons, two with PNH and two with aHUS, had inpatient claims for both meningitis and sepsis NOS during their risk period, including three that had both diagnoses on the same day or one day apart; the other person had separate claims of meningitis NOS and sepsis NOS close to two years apart, though both occurred during eculizumab treatment. All four individuals had information on potential etiologies in their inpatient claims: meningitis due to bacteria NOS (n = 2), pneumococcal meningitis and pneumococcal sepsis (n = 1), and viral meningitis (n = 1).

## Discussion

In our cohort of commercially insured individuals who received eculizumab between 2007 and 2017, most patients appeared to have aHUS or PNH, as expected. The age and sex distribution and many underlying conditions we observed in this cohort were consistent with previous literature on the characteristics of patients with aHUS and PNH [14–21]. However, 20% of eculizumab recipients in our cohort had an undetermined indication; 68.4% of these only received one eculizumab injection. Only 45.3% were up-to-date for MenACWY booster doses and 27.7% had claims for MenB vaccine. Claims for long-term antibiotic use in our cohort were also uncommon with only 7.1% receiving antibiotics for ≥50% of their risk period.

We examined the co-morbidities of individuals with undetermined eculizumab indication to shed light on the possible reasons for eculizumab treatment. Claims for cancer and joint-related diagnoses around the time of the first documented eculizumab treatment were unexpectedly common among eculizumab recipients with unknown treatment indication and among those with aHUS or PNH. Additional evaluations using data sources with more detailed clinical and diagnostic information would be helpful to understand why individuals without ICD-9/10 codes for aHUS, PNH, or gMG might be receiving eculizumab.

Although meningococcal vaccines are recommended for eculizumab recipients, low frequency of MenACWY booster dose and MenB primary series claims in our cohort suggests that many eculizumab recipients are not up-to-date for these vaccines. In previous reports, most (89–95%) eculizumab recipients who developed meningococcal disease had received ≥1 meningococcal vaccination [6, 22, 23]. We were unable to assess whether individuals in our cohort had ever received a MenACWY vaccine, as we only had access to the claims from the time period included in the Marketscan database; however, our findings show that many had not received a vaccine in the previous five years and therefore were not up-to-date with recommended MenACWY booster doses. The reasons that many eculizumab recipients were not up-to-date with MenACWY boosters are unknown, but could include limited patient and/or provider awareness that regular MenACWY booster doses are recommended in this population.

There is less published information on MenB vaccine uptake in eculizumab recipients. In one report, 67% of eculizumab recipients who developed meningococcal disease since June 2015 had received ≥1 MenB vaccine dose; however, this report included only six patients who developed disease in this time frame [22]. Given that MenB vaccine was not licensed in the United States until 2014–2015, the low uptake observed in our analysis may reflect slow implementation of this recommendation and could increase over time. MenB uptake was higher among persons aged 16–23 years, potentially related to the recommendation for healthy adolescents in this age group to receive MenB vaccine based on shared clinical decision-making; however, reason for vaccination could not be assessed as this information was not available in the Marketscan database.

Only 7.1% of eculizumab recipients had claims for antibiotics for ≥50% of the period of increased risk for meningococcal disease, but this proportion increased from 0% in 2007 to 19.1% in 2017. The low overall proportion of individuals with claims for long-term antibiotics during eculizumab treatment is presumably related to the absence of recommendations on antibiotic use among eculizumab recipients in the United States. The proportion with claims for antibiotics for ≥50% of the risk period was higher among transplant recipients, who likely received antibiotics to prevent post-operative infection. The proportion of such antibiotic use was also higher for children aged <18 years, which may reflect increased caution for pediatric patients; however, this could also reflect more frequent bacterial infections among these patients resulting in frequent antibiotic treatment.

Our results provide information on current antibiotic use from claims in eculizumab recipients, but do not shed light on its effectiveness in preventing meningococcal disease during the period of increased risk. Currently no data are available on effectiveness of antibiotic prophylaxis for eculizumab recipients. However, our description of the current use of long-term antibiotics among eculizumab recipients can provide a foundation for planning potential analyses of antibiotic effectiveness in this population. Furthermore, our characterization of eculizumab recipients (including treatment duration) can inform what antibiotic prophylaxis strategies might be feasible and appropriate to consider.

In our cohort one person had claims for meningococcemia two months after completing eculizumab treatment, which is within the period of increased risk for meningococcal disease per the eculizumab package insert [1]. We also identified 47 persons with claims for meningitis and/or sepsis NOS during the risk period. Most had no additional information on potential etiology in their inpatient claims from the meningitis and/or sepsis hospitalization, so we were unable to ascertain whether meningococcal disease was suspected for any of these cases. Alternatively, if these cases were not caused by *Neisseria meningitidis*, they may indicate that eculizumab recipients might be at increased risk for other pathogens, either because of eculizumab use itself or because of underlying or co-morbid conditions common among persons receiving eculizumab. Additional analyses using data sources with more detailed diagnostic information and laboratory test results would shed light on the etiology and risk factors for these infections.

Our analysis is subject to several limitations. First, the IBM Marketscan Commercial Database is not representative of the entire U.S. population; for instance, persons with public insurance or who are uninsured are not included. We hope to explore the patient population of eculizumab recipients within public insurance databases in the future, when additional years of data are available. Second, this is a descriptive analysis of persons receiving eculizumab. Future analyses comparing eculizumab recipients to an appropriate comparison group of individuals not receiving eculizumab would be valuable to assess how vaccine and antibiotic use among eculizumab recipients differ from those in the general population. Third, only claims billed to insurance are included in the database, so any healthcare encounter, vaccination, and/or prescription paid for out-of-pocket would not be included. Fourth, as ICD codes are used for billing purposes and miscoding can occur, disease misclassification is possible. Fifth, the reasons for antibiotic prescription were not captured in the IBM Marketscan Commercial Database, so we could not ascertain whether antibiotics were given for meningococcal disease prophylaxis, treatment of other infections, or other purposes. Finally, laboratory results were unavailable to confirm or determine the serogroup of the meningococcemia case or determine what, if any, testing was performed to detect potential etiologies of meningitis or sepsis NOS cases.

Persons receiving eculizumab will be at increased risk for meningococcal disease for the duration of their treatment, which our findings demonstrate may vary from a single injection to many years. Currently, eculizumab recipients represent a small population in need of targeted meningococcal disease prevention strategies. However, licensed indications for eculizumab use have expanded [1] and many additional complement inhibitors are in development [24, 25], suggesting that the number of persons with an elevated risk for meningococcal disease due to complement inhibitor use will continue to increase. Consequently, evaluating current and potential meningococcal disease prevention strategies is crucial for this population. By describing the population receiving eculizumab and the current meningococcal disease prevention strategies used, we hope to provide a foundation for further investigation of how patients receiving complement inhibitors can best be protected from meningococcal disease.

## Supporting information

S1 Table Claim codes used to define eculizumab, meningococcal vaccination, or antibiotics. (DOCX)

S2 Table International Classification of Diseases (ICD) 9/10 codes used to define medical conditions and their description. (DOCX)

S3 Table Co-morbidities and potential etiology among eculizumab recipients diagnosed with meningococcal disease, meningitis not otherwise specified and/or sepsis not otherwise specified in the IBM Marketscan Commercial Database, 2007–2017. (DOCX)

S1 Fig Total number of persons receiving ≥1 dose eculizumab each year by indication, IBM Marketscan Commercial Database, 2007–2017. (TIF)

S2 Fig Median duration of eculizumab treatment among (a) all eculizumab recipients in the IBM Marketscan Commercial Database and (b) by indication, 2007–2017.
(TIF)

S3 Fig Receipt of a) at least one MenACWY vaccine in the five years prior to the last documented eculizumab dose among eculizumab recipients and b) one or more doses or complete series (2–3 doses) of MenB vaccine, IBM Marketscan Commercial Database, 2007–2017.
(TIF)
