# Supplementary material for: Characteristics of and meningococcal disease prevention strategies for commercially insured persons receiving eculizumab in the United States
Source: PLoS One. 2020 Nov 12;15(11):e0241989. doi: 10.1371/journal.pone.0241989 (PMC7660549; doi:10.1371/journal.pone.0241989)
Supplement: S1 Table — (DOCX) [file pone.0241989.s001.docx]

S1 Table. Claim codes used to define eculizumab, meningococcal vaccination, or antibiotics.

| Medical condition, meningococcal vaccination, or antibiotic | Code Type | Code | Description of code |
| --- | --- | --- | --- |
| Eculizumab | Healthcare Common Procedure Coding System (HCPCS) | J1300 | Injection, eculizumab |
|  | HCPCS | C9236 | Injection, eculizumab |
|  | National Drug Codes (NDC) | 25682-0001-01 | Injection, eculizumab |
| Meningococcal vaccine containing serogroups A, C, W, and/or Y | Current Procedural Terminology (CPT) | 90644 | Meningococcal conjugate vaccine, serogroups C & Y and *Haemophilus influenzae* type b vaccine (Hib-MenCY), 4 dose schedule, when administered to children 6 weeks-18 months of age, for intramuscular use |
|  | CPT | 90733 | Meningococcal polysaccharide vaccine, serogroups A, C, Y, W-135, quadrivalent (MPSV4), for subcutaneous use |
|  | CPT | 90734 | Meningococcal conjugate vaccine, serogroups A, C, Y and W-135, quadrivalent (MCV4 or MenACWY), for intramuscular use |
|  | HCPCS | G9414 | Patient had one dose of meningococcal vaccine on or between the patient's 11th and 13th birthdays |
|  | NDC | 46028-0208, 58160-0955-09, 58160-0958-01, 58160-0959-01, 58160-0955 | MenACWY (Menveo) |
|  | NDC | 49281-0489-01, 49281-0489-91, 49281-0489, 49281-0488-95, 49281-0488-91 | MenACWY (Menactra) |
|  | NDC | 49281-0487-58, 49281-0488-78, 49281-0489-05, 49281-0489-95, 49281-0489-10, 49281-0489-04, 49281-0466-08, 49281-0466-31, 49281-489, 54569-3188-00, 54569-4840-00 | Meningococcal polysaccharide (MPSV4, MenomuneACWY) |
|  | NDC | 58160-801-11, 58160-0809-05, 58160-0809-01, 58160-0809, 58160-0801-11 | Meningococcal polysaccharide AC, Hib-MenCY (Menhibrix) |
| Serogroup B meningococcal vaccine | CPT | 90620 | Meningococcal recombinant protein and outer membrane vesicle vaccine, serogroup B (MenB-4C), 2 dose schedule, for intramuscular use |
|  | CPT | 90621 | Meningococcal recombinant lipoprotein vaccine, serogroup B (MenB-FHbp), 2 or 3 dose schedule, for intramuscular use |
|  | NDC | 00005-0100-01, 00005-0100-05, 00005-0100-10, 00005-0100-02 | MenB-FHbp |
|  | NDC | 46028-0114-01, 46028-0114-02, 46028-0114-11, 46028-0114, 58160-0976-06, 58160-0976-20, 58160-0976-02, 58160-0976 | MenB-4C |
| Macrolides (based on 9-digit substring) | NDC | 000030160, 000030161, 000053061, 000053119, 000053120, 000053250, 000053251, 000053700, 000053706, 000053732, 000070367, 000070369, 000080555, 000080556, 000080576, 000080578, 000090939, 000090940, 000158367, 000220695, 000220715, 000240490, 000318317, 000440207, 000440208, 000470672, 000470919, 000472330, 000472331, 000472545, 000491590, 000540036, 000540037, 000633006, 000690400, 000693050, 000693051, 000693060, 000693070, 000693080, 000693110, 000693120, 000693130, 000693140, 000693150, 000694170, 000710663, 000710696, 000740202, 000740205, 000740206, 000740207, 000740211, 000740648, 000742586, 000742589, 000743163, 000743165, 000743188, 000743246, 000743247, 000743368, 000743389, 000743494, 000743747, 000743748, 000745729, 000746227, 000746290, 000746301, 000746302, 000746303, 000746304, 000746305, 000746306, 000746308, 000746314, 000746316, 000746320, 000746321, 000746326, 000746342, 000746346, 000746360, 000746365, 000746368, 000746369, 000746371, 000746373, 000746476, 000746478, 000746481, 000746482, 000746483, 000747156, 000748030, 000840743, 000882223, 000882225, 000930775, 000932026, 000932027, 000937146, 000937147, 000937148, 000937149, 000937157, 000937158, 000937169, 000937244, 000945106, 001021090, 001021091, 001022725, 001022825  001432110, 001441348, 001502458, 001502459, 001502462, 001502464, 001570546, 001570856, 001570857, 001722458, 001722823, 001820538, 001820539, 001821227, 001821332, 001821371, 001821398, 001821402, 001821404, 001821489, 001821530, 001821560, 001821567, 001821773, 001827063, 001850275, 001857203, 001857206, 001857209, 001857212, 001870253, 002052848, 002052849, 002230912, 002230914, 002236140, 002236141, 002236142, 002236143, 002282184, 002282186, 002282189, 002282197, 002282204, 002282283, 002282553, 002526032, 002526033, 003022710, 003022725, 003022730, 003022731, 003022732, 003022740, 003022762, 003022763, 003040121, 003040122, 003040124, 003040590, 003040714, 003040715, 003040969, 003040970, 003042011, 003042064, 003491010, 003491011, 003498016, 003498227, 003498303, 003498384, 003498403, 003498806, 003498835, 003498881, 003498913, 003498974, 003590185, 003590186, 003590187, 003640530, 003642005, 003642031, 003642038, 003642057, 003642067, 003642070, 003642074, 003642078, 003642079, 003642319, 003642425, 003642494, 003672204, 003682015, 003682018, 003682019, 003683019, 003684052, 003780106, 003780107, 003781533, 003781534, 003781535, 003786400, 003788250, 003788500, 003852015, 003852016, 004030140, 004030141, 004030142, 004030145, 004030146, 004030147, 004030148, 004030313, 004030821, 004030825, 004030826, 004030831, 004030832, 004030835, 004030844, 004030845, 004030846, 004031040, 004031042, 004031613, 004031615, 004031617, 004032719, 004033563, 004034187, 004034621, 004034623, 004034625, 004034869, 004035151, 004052730, 004052735, 004054395, 004054396, 004054398, 004054399, 004054401, 004054406, 004054411, 004054412, 004090144, 004096476, 004096478, 004096482, 004181660, 004182304, 004270144, 004300696, 004510490, 004635012, 004691660, 004691663, 004691670, 004720971, 004720974, 004720977, 004720979, 004900056, 005240208, 005270784, 005271061, 005271931, 005271932, 005360050, 005360250, 005360265, 005360310, 005360318, 005360322, 005360335, 005360337, 005360351, 005360354, 005360360, 005360368, 005360371, 005361273, 005361276, 005372096, 005372098, 005372241, 005376376, 005550013, 005550215, 005550219, 005550230, 005550235, 005550236, 005550259, 005550445, 005550495, 005550532, 005550533, 005550584, 005800145, 005800146, 005800477, 005800496, 005800499, 005800672, 005800870, 005801152, 005801153, 005801707, 005801708, 005801709, 005912805, 005915479, 006031202, 006031203, 006031206, 006031207, 006033510, 006033514, 006033548, 006033551, 006033552, 006033553, 006033554, 006036563, 006150124, 006150143, 006150168, 006150169, 006150180, 006412304, 006412309, 006412313, 006770343, 006770344, 006770608, 006770653, 006770673, 006770806, 006770838, 006770839, 006771033, 006771303, 006771381, 006860106, 006860107, 006860965, 006860970, 006860977, 006860979, 007039085, 007039089, 007191332, 007191335, 007191336, 007191337, 007191338, 007191339, 007191340, 007194358, 007194359, 007194360, 007194365, 007197051, 007197370, 007770303, 007770807, 007770809, 007771404, 007771409, 007771441, 007772105, 007772125, 007772126, 007772303, 007772315, 007772317, 007772318, 007790376, 007790381, 007790382, 007790383, 007790384, 007790386, 007790387, 007790388, 007811491, 007811496, 007811497, 007811820, 007811850, 007811873, 007811941, 007811961, 007811962, 007812070, 007815776, 007815789, 007815793, 007816022, 007816023, 007817043, 008142970, 008142975, 008142980, 008142986, 008142988, 008142990, 008142995, 008143002, 008200117, 008200118, 008351121, 008352121, 008391293, 008395079, 008395185, 008396362, 008396387, 008396482, 008396568, 008396588, 008396713, 008396714, 008397514, 008397602, 008397656, 008397660, 008790105, 009042458, 009042459, 009042462, 009042463, 009042464, 009042465, 009042468, 009042470, 009042471, 009042472, 009042474, 009042475, 009042653, 009046010, 009046011, 009046405, 009047743, 009170162, 009950240, 009950280, 100190648, 105440522, 105440594, 105440595, 105440596, 105440952, 107540548, 107540549, 118450219, 118450234, 118450235, 118450421, 118450422, 118450476, 118450488, 119170200, 119170210, 120710008, 120710009, 120710336, 120710418, 120710482, 120710483, 120710484, 120710493, 122800057, 122800064, 122800101, 122800109, 122800112, 122800260, 122800268, 122800269, 125350063, 134110131, 134110185, 165900035, 165900088, 165900089, 165900091, 165900248, 165900362, 165900382, 165900383, 165900427, 170224102, 170224123, 172360064, 172360152, 172360154, 172360155, 172360405, 172360760, 172360783, 172360784, 172360785, 172360786, 172360972, 174780307, 188370338, 188370340, 188370356, 216950012, 216950013, 216950116, 216950387, 216950388, 216950389, 216950444, 216950457, 216950548, 216950549, 216950557, 216950558, 216950690, 234905329, 234905330, 234905331, 234905503, 234905504, 234905505, 234905509, 234905512, 234905513, 234905516, 234905517, 234905522, 234905523, 234905526, 234906904, 234906905, 234907758, 234907760, 242000628, 242080965, 242080970, 243380100, 243380102, 243380104, 243380106, 243380110, 243380112, 243380114, 243380120, 243380122, 243380124, 243380126, 243380130, 243380132, 243380134, 243380136, 250210112, 313570040, 332610139, 332610402, 332610403, 332610443, 332610472, 332610618, 332610631, 332610736, 332610808, 332610879, 332610919, 333580040, 333580041, 333580086, 333580087, 333580132, 333580133, 333580135, 333580136, 333580367, 333580368, 333580377, 353560017, 353560025, 353560044, 353560128, 353560199, 353560487, 353560704, 353560798, 353560827, 354700013, 354700107, 354700165, 354700262, 354700263, 354700322, 354700323, 354701156, 354701157, 417640110, 417640159, 417640160, 422540188, 422540199, 422540200, 422910263, 430630090, 430630110, 430630506, 430630520, 430630524, 430630530, 430630540, 430630568, 430630572, 430630713, 430630728, 430630752, 430630823, 433860470, 433860471, 437970288, 455650006, 470280013, 472021218, 472021237, 472021239, 472021240, 472021259, 472021260, 472021328, 472021409, 472021421, 472022053, 472022319, 472022381, 472022382, 476790587, 476790588, 476790755, 476790811, 476790812, 482530594, 482530595, 486520430, 497270154, 497270174, 497270175, 497270234, 497270582, 497270583, 497270600, 497270601, 499990012, 499990014, 499990023, 499990067, 499990080, 499990096, 499990101, 499990260, 499990315, 499990407, 499990515, 499990516, 499990582, 499990743, 499990786, 499990810, 499990904, 500900339, 500900361, 500900958, 500900960, 500900983, 500900984, 500900994, 500901410, 500901655, 500901788, 500901830, 500901835, 500901868, 500901939, 500902334, 500902335, 500902407, 500902441, 500902491, 500902509, 500902515, 500903193, 500903213, 500903298, 500903385, 501110767, 501110787, 501110788, 501110789, 501110791, 501110792, 501110793, 501110794, 502620098, 502680098, 502680099, 502680100, 502680101, 502680103, 502680104, 502680178, 502680179, 504360787, 504361496, 504364356, 504364357, 510790040, 510790361, 510790362, 510790492, 510790591, 510790671, 510790672, 510790673, 512850445, 513090306, 513090307, 513090308, 514320170, 514320171, 514320173, 514320174, 514320175, 514320177, 514320178, 514320179, 514320581, 514320582, 514320584, 514320591, 514320693, 514320826, 516410111, 516550098, 516550120, 516550152, 516550297, 516550480, 516550519, 516550674, 516550701, 517280139, 517280140, 517280141, 517280163, 517280164, 517280565, 517280566, 517280604, 518620194, 520150080, 521890514, 524060632, 524060633, 524060634, 525360103, 525360105, 525360134, 525360136, 529590057, 529590058, 529590060, 529590061, 529590062, 529590063, 529590064, 529590164, 529590230, 529590240, 529590242, 529590303, 529590304, 529590305, 529590310, 529590313, 529590339, 529590341, 529590427, 529590442, 529590504, 529590505, 529590657, 529590816, 529590836, 529590838, 529590927, 529590932, 529591052, 529591053, 529591055, 529591409, 529591423, 529591424, 529591452, 532170032, 532170160, 532170161, 532170173, 532170263, 532580121, 532580122, 532580125, 532581660, 532581663, 532581670, 532585729, 532586301, 532586304, 532586316, 532586320, 532586321, 532586346, 532610526, 532610535, 532610536, 532610546, 534890274, 534890275, 534890276, 534890277, 534890278, 534890279, 535060308, 535060309, 535060310, 535060375, 535060381, 539785029, 542740428, 542740429, 542740562, 542740578, 542740579, 542740580, 545380140, 545380240, 545380340, 545380440, 545690123, 545690124, 545690125, 545690126, 545690127, 545690128, 545690129, 545690131, 545690132, 545690134, 545690135, 545690157, 545691026, 545691890, 545692000, 545692001, 545692002, 545692132, 545692187, 545692199, 545692281, 545692433, 545692454, 545692502, 545692507, 545692508, 545692699, 545692721, 545692746, 545692821, 545692950, 545692952, 545693039, 545693057, 545693318, 545693347, 545693356, 545693439, 545693460, 545693461, 545693531, 545693541, 545693556, 545693563, 545693631, 545693896, 545693897, 545694163, 545694230, 545694231, 545694232, 545694270, 545694271, 545694417, 545694497, 545694522, 545694567, 545694681, 545694953, 545695038, 545695039, 545695042, 545695043, 545695175, 545695375, 545695448, 545695587, 545695688, 545695698, 545695754, 545695755, 545695756, 545695771, 545695804, 545695806, 545695807, 545695808, 545695809, 545695810, 545695829, 545696083, 545696084, 545696204, 545696353, 545696448, 545696476, 545697053, 545697055, 545698021, 545698313, 545698350, 545698355, 545698855, 545698867, 548680018, 548680220, 548680224, 548680225, 548680302, 548680333, 548680413, 548680654, 548680840, 548680914, 548680971, 548680985, 548681146, 548681230, 548681231, 548681386, 548681387, 548681405, 548681426, 548681774, 548681865, 548681886, 548681887, 548682190, 548682338, 548682840, 548683233, 548683244, 548683384, 548683648, 548683820, 548684076, 548684077, 548684078, 548684183, 548684191, 548684527, 548684644, 548684919, 548685171, 548685404, 548685430, 548685471, 548685478, 548685487, 548685647, 548685648, 548685938, 549770106, 549770114, 549770115, 549770116, 550451113, 550451168, 550451270, 550451381, 550451442, 550451471, 550451865, 550452012, 550452076, 550452162, 550452372, 550452373, 550452492, 550452942, 550453442, 550453452, 550453466, 550453479, 550453490, 550453693, 550453698, 550453725, 550453726, 550453727, 550453922, 550480072, 550480254, 550810209, 550810221, 550810222, 550810223, 550810224, 550810225, 550810226, 550810761, 550810897, 550840035, 550840037, 550840038, 550840039, 550840042, 551500174, 551750311, 551750812, 551751016, 551751502, 551751753, 551751760, 551751805, 551751806, 551752009, 551752111, 551752196, 551752199, 551752205, 551752213, 551752521, 551752677, 551752680, 551752741, 551754269, 551754271, 551754399, 551754408, 551754427, 551754428, 551755219, 551755220, 551755225, 551755226, 552890004, 552890008, 552890021, 552890025, 552890027, 552890050, 552890075, 552890110, 552890111, 552890112, 552890120, 552890217, 552890274, 552890310, 552890426, 552890525, 552890645, 552890705, 552890813, 552890909, 552890915, 552890964, 557000050, 557000065, 557000206, 557000211, 557000351, 557000432, 557000535, 558870033, 558870071, 558870138, 558870147, 558870311, 558870332, 558870333, 558870372, 558870586, 558870746, 558870747, 558870768, 558870785, 558870787, 558870813, 558870849, 558870855, 558870933, 558870955, 561260387, 561260391, 561260397, 572370044, 572370045, 578660250, 578660261, 578660351, 578660446, 578663136, 578664356, 578667071, 578668001, 580160086, 580160123, 580160124, 580160125, 580160126, 580160127, 580160128, 580160145, 580160162, 580160167, 580160175, 580160176, 580160177, 580160184, 580160299, 580160362, 580160380, 580160391, 580160550, 580161017, 580161018, 580161023, 580161024, 580161025, 580161036, 580161037, 580161038, 580161039, 580161235, 580161835, 580161845, 580161853, 580164113, 580164114, 580164814, 580164847, 588640067, 588640195, 588640199, 588640200, 588640615, 588640632, 588640655, 588640791, 588640837, 588640845, 597460742, 597460743, 597623051, 597623060, 597623070, 597623080, 597623110, 597623120, 597623130, 597623140, 603460009, 603460017, 603460021, 603460025, 603460032, 603460037, 603460065, 603460077, 603460088, 603460098, 603460108, 603460122, 603460195, 603460211, 603460325, 603460351, 603460358, 603460413, 603460429, 603460440, 603460445, 603460580, 603460583, 603460597, 603460601, 603460617, 603460630, 603460642, 603460643, 603460644, 603460645, 603460646, 603460653, 603460670, 604290070, 605052581, 605052582, 605052583, 605052615, 605052616, 605056076, 606870271, 606870282, 606870314, 607600261, 607600320, 613920113, 619190096, 619190362, 619190382, 619190555, 619190643, 619190776, 620370777, 625590440, 626821022, 627560512, 631870007, 631870169, 631870284, 631870376, 631870604, 633040725, 633040726, 633040821, 633040822, 633230398, 636291284, 636291374, 636291834, 636291835, 636291883, 636292673, 636292685, 636292755, 636292887, 636292938, 636292940, 637390575, 638740110, 638740127, 638740129, 638740130, 638740156, 638740159, 638740160, 638740231, 638740246, 638741197, 646790949, 646790954, 646790961, 646790962, 646790964, 658620225, 658620226, 658620641, 658620642, 661050507, 661050653, 661050670, 661050743, 661050744, 661160163, 661160243, 661160274, 661160279, 662670086, 662670088, 662670089, 662670090, 662670091, 662670927, 662670928, 663360017, 663360037, 663360077, 663360098, 663360108, 663360168, 663360347, 663360400, 663360429, 663360466, 663360580, 663360617, 663360644, 663360645, 663360846, 680710337, 680710392, 680711323, 680711527, 680711528, 680711584, 680840278, 680840279, 680840437, 680840443, 680840464, 680840651, 680840656, 680840906, 680840913, 680840920, 681150047, 681150048, 681150126, 681150131, 681150132, 681150133, 681150134, 681150135, 681150136, 681150364, 681150445, 681150541, 681150931, 681800160, 681800161, 681800162, 682581975, 682581976, 682583012, 682583051, 683080250, 683820761, 683820762, 683820763, 683820764, 683820765, 683870548, 683870565, 687740120, 687740122, 687740302, 687740303, 692381484, 692381485, 696770129, 707101457, 707101458, 707101459, 707101460, 708600100, 708820107, 708820108, 708820118, 712050021, 712050119, 713350017, 713350795, 999990001, 999990009, 999990011, 999990012, 999990020, 999990033, 999990040, 999990203, 999991011, 999992222, 999992223, 999992224, 999993652, 999996545, 999998751 | Oral macrolides |
| Penicillin (based on 9-digit substring) | NDC | 000020327, 000020329, 000020346, 000021403, 000021405, 000021406, 000021410, 000022307, 000022316, 000027147, 000027185, 000027204, 000027205, 000030101, 000030109, 000030115, 000030116, 000030122, 000030134, 000030164, 000030165, 000030168, 000030299, 000030309, 000030634, 000030668, 000030673, 000030681, 000030682, 000030734, 000030735, 000030743, 000030746, 000030969, 000030972, 000031616, 000031737, 000031738, 000032710, 000032711, 000032712, 000032713, 000032714, 000032715, 000032911, 000032912, 000032913, 000032915, 000032916, 000032917, 000032918, 000032919, 000032920, 000032921, 000032987, 000032989, 000032990, 000032991, 000032992, 000032993, 000041960, 000041961, 000053135, 000053136, 000053144, 000053145, 000053146, 000053147, 000053255, 000053586, 000053587, 000053588, 000053589, 000053779, 000053780, 000053865, 000053866, 000053870, 000053871, 000053874, 000053875, 000070101, 000070102, 000070103, 000070104, 000070106, 000070109, 000070111, 000070112, 000070113, 000070114, 000070116, 000070117, 000070118, 000070119, 000080004, 000080018, 000080021, 000080026, 000080036, 000080053, 000080054, 000080055, 000080057, 000080058, 000080059, 000080077, 000080079, 000080163, 000080165, 000080176, 000080255, 000080272, 000080309, 000080315, 000080360, 000080361, 000080390, 000080415, 000080431, 000080464, 000080513, 000080557, 000080558, 000080559, 000080560, 000080593, 000080599, 000080600, 000080614, 000080615, 000080751, 000082517, 000090586, 000090671, 000090787, 000090865, 000157100, 000157101, 000157102, 000157103, 000157195, 000157196, 000157224, 000157225, 000157226, 000157276, 000157277, 000157278, 000157279, 000157300, 000157401, 000157402, 000157403, 000157404, 000157405, 000157496, 000157506, 000157507, 000157508, 000157509, 000157607, 000157658, 000157805, 000157808, 000157856, 000157884, 000157892, 000157893, 000157935, 000157941, 000157961, 000157964, 000157965, 000157970, 000157977, 000157978, 000157979, 000157981, 000157982, 000157985, 000157988, 000157992, 000157993, 000157998, 000220428, 000220430, 000220432, 000220434, 000220783, 000220809, 000220820, 000220901, 000220927, 000220928, 000220970, 000220971, 000268211, 000268212, 000268213, 000268214, 000268220, 000268311, 000268312, 000268314, 000296004, 000296005, 000296006, 000296007, 000296008, 000296009, 000296010, 000296015, 000296020, 000296025, 000296028, 000296030, 000296032, 000296035, 000296038, 000296044, 000296045, 000296046, 000296047, 000296048, 000296049, 000296071, 000296072, 000296073, 000296074, 000296075, 000296080, 000296085, 000296086, 000296087, 000296090, 000296092, 000296094, 000296096, 000296150, 000296160, 000296165, 000296170, 000296200, 000296204, 000296205, 000296210, 000296212, 000296250, 000296255, 000296351, 000296352, 000296370, 000296372, 000296374, 000296376, 000296400, 000296402, 000296405, 000296406, 000296407, 000296550, 000296552, 000296555, 000296558, 000296559, 000296571, 000296579, 000296595, 000296600, 000296605, 000296610, 000296612, 000296613, 000296615, 000296620, 000296625, 000296630, 000296681, 000296682, 000296683, 000296684, 000296685, 000317105, 000317107, 000317117, 000317127, 000318205, 000318207, 000318217, 000318227, 000460661, 000460662, 000470038, 000470402, 000470404, 000470648, 000470673, 000470730, 000470731, 000470945, 000470946, 000470949, 000470950, 000472301, 000472302, 000472449, 000472500, 000472501, 000472506, 000472995, 000490013, 000490014, 000490022, 000490023, 000490024, 000490031, 000490032, 000490210, 000490350, 000490357, 000490420, 000490430, 000490510, 000490520, 000490530, 000490540, 000491310, 000491320, 000491410, 000491419, 000491430, 000560153, 000560154, 000560249, 000560250, 000631011, 000631012, 000631013, 000631048, 000631049, 000631093, 000631110, 000631111, 000631112, 000690026, 000690027, 000690029, 000690046, 000690065, 000690071, 000690210, 000712301, 000712302, 000712303, 000712449, 000712500, 000712506, 000714178, 000714179, 000840160, 000840161, 000840400, 000840410, 000930613, 000930615, 000931171, 000931172, 000931173, 000931174, 000932263, 000932264, 000932267, 000932268, 000932270, 000932272, 000932274, 000932275, 000932277, 000932279, 000933107, 000933109, 000933111, 000933113, 000933115, 000933117, 000933119, 000933121, 000933123, 000933125, 000934125, 000934127, 000934150, 000934155, 000934157, 000934159, 000934160, 000934161, 000935145, 000935146, 000935194, 000935195, 000935198, 000935199, 000938055, 000938675, 001021010, 001021011, 001021012, 001021013, 001021014, 001021015, 001021200, 001021205, 001021210, 001021211, 001021220, 001021221, 001021800, 001021802, 001021805, 001021810, 001021815, 001021820, 001021822, 001021825, 001431385, 001431390, 001431395, 001431400, 001432270, 001433062, 001433063, 001439249, 001439285, 001439836, 001439837, 001439853, 001439886, 001439887, 001439888, 001439889, 001439938, 001439939, 001439951, 001439981, 001439982, 001441210, 001441212, 001441475, 001441479, 001444220, 001444230, 001444240, 001502017, 001502073, 001502428, 001502450, 001502451, 001504001, 001504004, 001504010, 001504014, 001570413, 001570414, 001570532, 001570533, 001570540, 001570541, 001570574, 001570575, 001727401, 001727402, 001727403, 001727404, 001727405, 001727406, 001727407, 001727408, 001727411, 001727414, 001727416, 001727417, 001727418, 001727419, 001727420, 001810601, 001820107, 001820115, 001820116, 001820121, 001820163, 001820274, 001820275, 001820276, 001820308, 001820540, 001820641, 001820674, 001820869, 001820898, 001821070, 001821071, 001821072, 001821073, 001821340, 001821341, 001821358, 001821359, 001821506, 001821507, 001821537, 001821720, 001821962, 001827069, 001870125, 001870127, 001910025, 002062100, 002062404, 002062405, 002062408, 002062409, 002062411, 002062413, 002062416, 002062501, 002063100, 002063501, 002063877, 002063879, 002063880, 002063882, 002064100, 002064501, 002065501, 002068452, 002068454, 002068455, 002068620, 002068820, 002068821, 002068822, 002068852, 002068854, 002068855, 002068859, 002068860, 002068861, 002068862, 002090050, 002090100, 002090150, 002090200, 002090250, 002090300, 002090350, 002090400, 002090450, 002096900, 002096950, 002097000, 002097100, 002097150, 002097250, 002097950, 002098000, 002098050, 002098100, 002098150, 002098300, 002098570, 002098574, 002098578, 002098580, 002098586, 002172825, 002230190, 002230191, 002282112, 002282114, 002282115, 002282116, 002282117, 002282119, 002282296, 002282300, 002282304, 002282324, 002282328, 002282330, 002282443, 002282444, 002282639, 002282640, 002282688, 002282689, 002526004, 002526006, 002526007, 002526020, 002526021, 002526026, 002526027, 002761230, 003003702, 003020260, 003020262, 003020265, 003020267, 003020270, 003020272, 003020275, 003020277, 003021110, 003021111, 003021700, 003021702, 003024290, 003024292, 003024900, 003024901, 003024910, 003024912, 003024914, 003024916, 003024922, 003040018, 003040019, 003040020, 003040021, 003040205, 003040206, 003040207, 003040208, 003040209, 003040210, 003040584, 003040585, 003040586, 003040587, 003040588, 003040589, 003040591, 003040695, 003040696, 003040708, 003040716, 003040731, 003040826, 003040827, 003040904, 003042043, 003042090, 003042091, 003321117, 003321121, 003321123, 003321172, 003321173, 003322207, 003322208, 003322267, 003322268, 003323107, 003323109, 003323111, 003323113, 003323115, 003323119, 003323121, 003323123, 003324115, 003324117, 003324125, 003324127, 003324129, 003324131, 003324140, 003324150, 003324154, 003324157, 003324159, 003381013, 003381015, 003381017, 003381019, 003381021, 003381023, 003381025, 003490190, 003490997, 003490998, 003490999, 003491003, 003491006, 003491009, 003491012, 003491014, 003491016, 003491021, 003491022, 003491023, 003491024, 003491025, 003491026, 003491050, 003491051, 003498224, 003498225, 003498226, 003498236, 003498319, 003498320, 003590015, 003590016, 003590067, 003590068, 003590069, 003590107, 003590114, 003590115, 003590116, 003590117, 003590118, 003590119, 003590153, 003590154, 003590277, 003590278, 003590282, 003590283, 003591006, 003591106, 003591107, 003591115, 003591116, 003640856, 003642001, 003642002, 003642003, 003642004, 003642010, 003642011, 003642014, 003642015, 003642020, 003642021, 003642022, 003642023, 003642024, 003642040, 003642041, 003642058, 003642059, 003642060, 003642061, 003642062, 003642064, 003642071, 003642569, 003642906, 003642908, 003647196, 003647215, 003647216, 003672112, 003672114, 003672300, 003672304, 003672324, 003672328, 003681180, 003780111, 003780112, 003780113, 003780114, 003780115, 003780116, 003780117, 003780118, 003780120, 003780121, 003780122, 003780123, 003780198, 003780204, 003780205, 003780206, 003780207, 003780273, 003780830, 003780831, 003780832, 003780833, 003780834, 003780835, 003780836, 003781204, 003781205, 003782115, 003782116, 003852013, 003852014, 003852040, 003853025, 003853026, 004030136, 004030138, 004030242, 004030244, 004030253, 004030254, 004030256, 004030259, 004030260, 004030263, 004030264, 004030265, 004030266, 004030267, 004030268, 004030269, 004030270, 004030271, 004030272, 004030273, 004030274, 004030275, 004030276, 004030277, 004030279, 004030357, 004030359, 004030361, 004030363, 004030365, 004030367, 004030369, 004030404, 004030455, 004030713, 004031620, 004031623, 004031624, 004031625, 004031626, 004031627, 004031628, 004031629, 004031630, 004032034, 004032733, 004034045, 004034289, 004034343, 004034709, 004035022, 004035183, 004052225, 004052250, 004052275, 004052300, 004052530, 004053500, 004053525, 004054083, 004054084, 004054086, 004054089, 004054090, 004054255, 004054256, 004054322, 004054323, 004054726, 004054727, 004054762, 004054763, 004054768, 004054769, 004054774, 004092687, 004092689, 004092987, 004092988, 004092998, 004092999, 004093374, 004093378, 004093379, 004093383, 004093385, 004093390, 004093713, 004093714, 004093715, 004093718, 004093719, 004093720, 004093725, 004093726, 004270135, 004270136, 004270138, 004270433, 004407101, 004407105, 004407108, 004407381, 004635005, 004635006, 004635011, 004635013, 004635014, 004635015, 004635016, 004635017, 004635018, 004635019, 004635020, 004635023, 004851170, 004910048, 005270565, 005270583, 005270835, 005270836, 005271097, 005271141, 005271239, 005360010, 005360016, 005360020, 005360030, 005360070, 005360080, 005360090, 005360097, 005360105, 005360170, 005360809, 005360908, 005361000, 005361101, 005361110, 005361120, 005361130, 005361140, 005361150, 005361160, 005361170, 005361180, 005361190, 005361300, 005361550, 005361551, 005361552, 005361571, 005362520, 005362527, 005362530, 005362537, 005362538, 005362540, 005362560, 005372011, 005372012, 005372135, 005372136, 005372137, 005372138, 005372139, 005372142, 005372143, 005372258, 005372315, 005372316, 005372318, 005372319, 005372320, 005372322, 005372764, 005372768, 005372769, 005372774, 005372775, 005372776, 005372777, 005376049, 005376151, 005376152, 005376160, 005376161, 005376162, 005376163, 005376284, 005376285, 005376408, 005376409, 005376504, 005376505, 005560049, 005800016, 005800017, 005800018, 005800019, 005800020, 005800021, 005800022, 005800023, 005800239, 005800240, 005800241, 005800242, 005800243, 005800244, 005800245, 005800246, 005800247, 005800282, 005800464, 005800465, 005800466, 005800467, 005800676, 005800677, 005801392, 005801544, 006032266, 006032267, 006032273, 006032274, 006032290, 006032291, 006033029, 006033030, 006033241, 006033242, 006034927, 006034928, 006035065, 006035067, 006035068, 006036500, 006036501, 006036520, 006036521, 006036584, 006036605, 006036606, 006150100, 006150101, 006150111, 006150113, 006150120, 006150121, 006150122, 006150123, 006150126, 006150127, 006412249, 006412251, 006412252, 006412253, 006412254, 006412255, 006412256, 006412257, 006412259, 006416116, 006416117, 006416118, 006416119, 006416120, 006416121, 006416123, 006590103, 006590104, 006590125, 006590130, 006596124, 006596134, 006770010, 006770011, 006770012, 006770013, 006770096, 006770097, 006770098, 006770104, 006770105, 006770107, 006770108, 006770109, 006770110, 006770411, 006770452, 006770453, 006770576, 006770660, 006770661, 006770928, 006770929, 006770930, 006770931, 006770932, 006770933, 006829112, 006829113, 006829114, 006829116, 006860600, 006860601, 006860602, 006860603, 006860608, 006860609, 006860610, 006860611, 006863107, 006863109, 006863111, 006863115, 006863117, 006863119, 006863121, 006863313, 006863702, 006863704, 006863708, 006863712, 006864140, 006864157, 006864159, 007038108, 007038118, 007038128, 007038129, 007038138, 007038139, 007038148, 007038149, 007038158, 007038208, 007038218, 007038219, 007038228, 007038229, 007038238, 007038308, 007038318, 007038319, 007038328, 007038329, 007038338, 007039095, 007039105, 007191070, 007191071, 007191080, 007191081, 007191228, 007191229, 007191282, 007191283, 007191655, 007191656, 007191690, 007191691, 007191695, 007191696, 007191697, 007191700, 007193300, 007197320, 007197321, 007197330, 007197331, 007197335, 007197350, 007197400, 007197450, 007197451, 007457007, 007457008, 007457021, 007457996, 007790032, 007790034, 007790045, 007790056, 007790057, 007790066, 007790067, 007790069, 007790070, 007790253, 007790254, 007790255, 007790294, 007790295, 007790699, 007790700, 007790769, 007790770, 007790771, 007790772, 007790775, 007790779, 007790780, 007797598, 007811098, 007811205, 007811615, 007811619, 007811643, 007811655, 007811831, 007811852, 007811874, 007811943, 007811985, 007812004, 007812006, 007812020, 007812034, 007812036, 007812144, 007812145, 007812220, 007812225, 007812248, 007812258, 007812555, 007812613, 007812999, 007813032, 007813033, 007813034, 007813094, 007813095, 007813099, 007813101, 007813103, 007813110, 007813113, 007813114, 007813124, 007813125, 007813126, 007813128, 007813129, 007813344, 007813350, 007813367, 007813400, 007813402, 007813404, 007813407, 007813408, 007813409, 007813412, 007813413, 007813700, 007813702, 007813704, 007813706, 007813708, 007813710, 007813712, 007813714, 007813716, 007813740, 007813742, 007813744, 007813746, 007813748, 007813750, 007813758, 007813760, 007813762, 007813764, 007813766, 007813768, 007814054, 007815060, 007815061, 007816039, 007816041, 007816102, 007816104, 007816120, 007816121, 007816135, 007816136, 007816139, 007816153, 007816156, 007816157, 007816191, 007816195, 007819109, 007819110, 007819111, 007819112, 007819113, 007819124, 007819125, 007819126, 007819210, 007819213, 007819224, 007819225, 007819242, 007819250, 007819261, 007819273, 007819344, 007819350, 007819367, 007819401, 007819402, 007819404, 007819407, 007819408, 007819409, 007819412, 007819413, 008140690, 008140692, 008140695, 008140700, 008140701, 008140720, 008140722, 008140725, 008140728, 008141752, 008141753, 008141754, 008142435, 008142436, 008145587, 008145818, 008145822, 008145863, 008145866, 008145870, 008145871, 008145875, 008145878, 008146315, 008351452, 008351453, 008351454, 008351457, 008351475, 008353000, 008353001, 008391724, 008391726, 008391727, 008391753, 008391766, 008395087, 008395130, 008395142, 008395144, 008395187, 008395188, 008395189, 008395190, 008396037, 008396038, 008396115, 008396116, 008396178, 008396393, 008396412, 008396413, 008396430, 008396436, 008396437, 008396445, 008396614, 008397277, 008397558, 008397559, 008397776, 008790125, 008790148, 008790149, 008790400, 009041519, 009041619, 009041649, 009042017, 009042073, 009042427, 009042428, 009042449, 009042450, 009042451, 009042452, 009042617, 009042618, 009042619, 009042620, 009042642, 009042643, 009042647, 009042648, 009042709, 009042710, 009044001, 009044004, 009044010, 009044014, 009047713, 009170180, 009170198, 009170343, 009485302, 009485303, 009485304, 009950311, 009950312, 009950450, 009950460, 009950470, 009950480, 009951040, 009951050, 009951060, 009951070, 009951080, 009951322, 009951417, 009953000, 009953100, 100030100, 100190630, 100190631, 100190632, 100190633, 100190634, 100190635, 100190636, 100190637, 100190638, 101390070, 101390071, 105170040, 105174000, 105440007, 105440018, 105440117, 105440119, 105440122, 105440296, 105440297, 105440331, 105440370, 105440434, 105440500, 105440546, 105440619, 105440896, 105440950, 105440951, 106310176, 106310565, 106310567, 107540583, 110420142, 113110870, 113110872, 115840381, 115840391, 115840401, 118450146, 118450147, 118450148, 118450149, 118450217, 118450218, 118450355, 118450356, 118450463, 118450464, 118450465, 118450466, 119160690, 119170037, 119170039, 119170040, 119170042, 119170680, 119170687, 119170688, 119170689, 119170690, 119170691, 119170692, 119170694, 120710007, 120710010, 120710011, 120710015, 120710016, 120710017, 120710073, 120710074, 120710078, 120710079, 120710244, 120710245, 120710246, 120710247, 120710291, 120710363, 120710369, 120710619, 120710620, 122800043, 122800089, 122800153, 122800154, 122800155, 122800156, 122800157, 122800158, 122800222, 125350058, 125350104, 125350108, 125350114, 125350170, 126340807, 127360078, 128300111, 128300114, 165900014, 165900015, 165900016, 165900017, 165900018, 165900019, 165900020, 165900076, 165900182, 165900183, 165900369, 165900399, 165900401, 165900424, 165900647, 167140234, 167140235, 167140292, 167140293, 167140294, 167140295, 167140296, 167140297, 167140298, 167140299, 167140476, 167140477, 167140478, 167140494, 170220282, 170220303, 170220324, 170220345, 170220396, 170220407, 170220496, 170220498, 170220500, 170220502, 170220504, 170220506, 170220962, 170220970, 170221979, 170221987, 170221991, 172360056, 172360057, 172360078, 172360111, 172360228, 172360230, 172360232, 172360234, 172360236, 172360319, 172360320, 172360321, 172360322, 172360332, 172360333, 172360500, 172360501, 172360502, 172360504, 172360505, 172360685, 188370042, 188370216, 188370268, 188370339, 188370357, 216950124, 216950214, 216950215, 216950294, 216950314, 216950315, 216950318, 216950384, 216950385, 216950401, 216950418, 216950544, 216950546, 216950559, 216950767, 216950772, 234900076, 234900081, 234905065, 234905066, 234905067, 234905068, 234905070, 234905071, 234905074, 234905075, 234905079, 234905081, 234905425, 234905426, 234906067, 234906068, 234906069, 234906070, 234906940, 234906977, 234906979, 234907220, 234907311, 234907312, 234907585, 234907950, 234907961, 242000652, 242000653, 242000654, 250210134, 250210135, 250210136, 250210137, 250210138, 250210139, 250210140, 250210141, 250210142, 250210143, 250210144, 250210146, 250210154, 250210162, 250210163, 250210164, 250210165, 250210166, 250210186, 250210187, 250210188, 253321030, 253322005, 253322017, 253324131, 332610111, 332610112, 332610119, 332610134, 332610136, 332610137, 332610138, 332610144, 332610167, 332610426, 332610428, 332610460, 332610462, 332610463, 332610464, 332610488, 332610600, 332610602, 332610726, 332610766, 332610949, 332610988, 332610989, 333580025, 333580026, 333580028, 333580029, 333580030, 333580031, 333580032, 333580033, 333580106, 333580107, 333580284, 333580285, 353560037, 353560188, 353560463, 353560981, 353560982, 353560983, 353560984, 353560985, 353560986, 353560989, 354700265, 354700266, 354700267, 354700268, 354701173, 354702017, 354702065, 354702066, 354702073, 354702427, 354702428, 354702446, 354702450, 354702476, 354703107, 354703109, 354703119, 354703121, 354704125, 354704127, 354704129, 354704131, 354704150, 354704155, 360000070, 360000071, 360000072, 360000073, 398220123, 398220125, 398220127, 398220139, 398220400, 398220600, 417640101, 417640102, 417640103, 417640108, 417640113, 417640114, 417640115, 417640116, 417640117, 417640123, 417640143, 417640144, 417640145, 417640146, 417640147, 417640148, 417640239, 417640240, 422540026, 422540120, 422540139, 422540181, 422540215, 422540232, 422540272, 422540282, 422910120, 422910121, 422910216, 425490531, 425490570, 425710160, 425710161, 425710162, 425820214, 430630015, 430630017, 430630054, 430630077, 430630096, 430630180, 430630341, 430630342, 430630377, 430630434, 430630473, 430630542, 430630557, 430630563, 430630574, 430630576, 430630704, 430630780, 430630820, 430630885, 435980003, 435980004, 435980006, 435980012, 435980020, 435980021, 435980203, 435980204, 435980205, 435980206, 435980207, 435980208, 435980209, 435980213, 435980218, 435980219, 435980220, 435980221, 435980222, 435980223, 435980224, 435980225, 437970051, 437970158, 437970159, 437970277, 437970278, 437970279, 437970280, 437970286, 437970287, 445670100, 445670101, 445670102, 445670103, 445670104, 445670210, 445670211, 445670212, 445670221, 445670222, 445670223, 445670311, 445670312, 445670801, 445670802, 445670803, 445670804, 445670882, 455650001, 455650002, 455650031, 455650032, 458650418, 458650526, 470280004, 470280053, 472021215, 472021233, 472021234, 472021253, 472021267, 472021270, 472021282, 472021287, 472021330, 472021333, 472021353, 472021354, 472021356, 472022005, 472022006, 472022098, 472022232, 472022233, 472022247, 472022248, 472022369, 472022370, 472022397, 472022422, 472022470, 472022471, 476790892, 476790893, 476790894, 476790895, 476790896, 476790897, 476790898, 476790899, 481020111, 482530141, 482530142, 482530144, 482530145, 482530160, 482530741, 482530742, 482530744, 482530754, 482530775, 486520428, 486520429, 486520432, 496480205, 496480206, 497270013, 497270014, 497270152, 497270155, 497270327, 497270328, 497270330, 497270332, 497270367, 497270368, 497270392, 497270393, 497270465, 497270466, 498840041, 498840053, 498840060, 498840072, 498840073, 498840142, 498840168, 498840201, 498840298, 498840299, 498840365, 498840568, 498840569, 498840570, 498840571, 498840574, 498840575, 498840576, 498840577, 498840578, 498840579, 498840580, 498840581, 498840623, 498840624, 498840625, 498840626, 498840627, 498840628, 498840629, 498840630, 498840631, 498840632, 498840633, 498840634, 499990001, 499990002, 499990015, 499990016, 499990033, 499990050, 499990117, 499990120, 499990168, 499990177, 499990191, 499990213, 499990246, 499990267, 499990332, 499990356, 499990365, 499990585, 499990651, 499990745, 499990766, 499990983, 500900116, 500900301, 500900302, 500900303, 500900304, 500900310, 500900404, 500900405, 500900406, 500900407, 500900408, 500900409, 500900411, 500900454, 500900455, 500900709, 500900711, 500900712, 500900713, 500900714, 500900821, 500900831, 500900832, 500900858, 500900928, 500900996, 500901016, 500901086, 500901347, 500901610, 500901612, 500901825, 500901826, 500901857, 500902042, 500902044, 500902251, 500902286, 500902287, 500902314, 500902547, 500903005, 500903047, 500903179, 500903187, 500903210, 500903293, 500903307, 500903309, 500903374, 500903425, 504360029, 504360031, 504360106, 504361001, 504363129, 504363613, 504364543, 504366051, 504367218, 509300107, 510790265, 510790267, 510790600, 510790601, 510790602, 510790603, 510790609, 510790610, 510790611, 510790612, 510790613, 510790615, 510790616, 512850840, 512850841, 514320018, 514320020, 514320022, 514320024, 514320102, 514320104, 514320318, 514320320, 514320328, 514320330, 514320332, 514320334, 514320493, 514320494, 514320655, 514320689, 514320690, 514320691, 514320692, 514320694, 514320695, 514320696, 514320697, 514320785, 516550009, 516550010, 516550011, 516550075, 516550104, 516550110, 516550156, 516550157, 516550158, 516550159, 516550160, 516550213, 516550258, 516550265, 516550630, 516550917, 517280137, 517280138, 517280245, 517280246, 517280247, 517280248, 517280322, 517280323, 517280324, 517280358, 517280359, 517280360, 517280361, 517280382, 517280383, 517280447, 517280448, 517280449, 517280507, 517280508, 517280509, 517280555, 517280620, 517280691, 524060516, 524060517, 524060519, 524060520, 524060543, 524060544, 524060599, 524060600, 524460706, 524460707, 524462273, 525550141, 525550142, 525550148, 525550149, 529590011, 529590012, 529590020, 529590021, 529590022, 529590048, 529590049, 529590130, 529590181, 529590209, 529590213, 529590246, 529590296, 529590333, 529590343, 529590389, 529590466, 529590468, 529590470, 529590478, 529590560, 529590613, 529590614, 529590615, 529590616, 529590617, 529590661, 529590702, 529590707, 529590793, 529590843, 529591005, 529591006, 529591012, 529591172, 529591425, 529591430, 529591431, 529591461, 529591462, 529591463, 532170078, 532170210, 532170217, 532170220, 532170249, 532170286, 532170290, 532170312, 532170314, 532170316, 532610544, 532610545, 532610547, 532610597, 534451070, 534451071, 534890168, 534890169, 534890170, 534890171, 534890172, 534890173, 534890174, 534890175, 534890219, 534890220, 534890249, 535060002, 535060301, 535060302, 535060311, 535060315, 535060348, 535060353, 535060354, 535060355, 535060374, 535060378, 535060795, 535060797, 535060799, 539781257, 539785002, 539785003, 539785018, 539785019, 539785042, 539785055, 542740426, 542740427, 542740485, 542740486, 542740487, 542740488, 542740489, 542740490, 542740491, 542740492, 542740493, 542740500, 542740501, 542740502, 542740503, 542740507, 542740508, 542740509, 542740510, 542740511, 542740512, 545380100, 545380110, 545380190, 545380200, 545380210, 545380290, 545690000, 545690080, 545690092, 545690093, 545690094, 545690096, 545690097, 545690098, 545690101, 545690107, 545690117, 545690120, 545690121, 545690122, 545690136, 545690137, 545690142, 545690143, 545690144, 545690145, 545690146, 545690152, 545690153, 545690298, 545690384, 545691019, 545691020, 545691022, 545691409, 545691508, 545691529, 545691530, 545691545, 545691546, 545691719, 545691746, 545691761, 545691789, 545691850, 545691859, 545691861, 545691880, 545691885, 545691889, 545691917, 545691959, 545691961, 545691962, 545691984, 545692033, 545692180, 545692200, 545692229, 545692316, 545692325, 545692380, 545692411, 545692702, 545692708, 545692710, 545692906, 545692907, 545692928, 545692929, 545692930, 545692931, 545692932, 545692933, 545692934, 545692935, 545692936, 545692937, 545692938, 545692939, 545692945, 545692953, 545692954, 545692991, 545693276, 545693277, 545693335, 545693457, 545693503, 545693689, 545693722, 545693876, 545693880, 545693986, 545694325, 545694327, 545694337, 545694338, 545694352, 545694353, 545694458, 545694592, 545694608, 545694790, 545694791, 545694796, 545694797, 545694889, 545694892, 545695002, 545695010, 545695011, 545695012, 545695031, 545695062, 545695182, 545695193, 545695314, 545695344, 545695470, 545695471, 545695487, 545695488, 545695499, 545695518, 545695522, 545695553, 545695622, 545695638, 545695689, 545695690, 545695727, 545695831, 545695876, 545695877, 545695957, 545695958, 545696037, 545696043, 545696401, 545696626, 545696754, 545697010, 545697024, 545697025, 545697026, 545698000, 545698001, 545698004, 545698300, 545698333, 545698334, 545698338, 545698341, 545698343, 545698354, 545698800, 545698853, 545698868, 548070251, 548680193, 548680195, 548680196, 548680199, 548680200, 548680286, 548680297, 548680348, 548680387, 548680388, 548680753, 548680807, 548680937, 548681018, 548681044, 548681171, 548681173, 548681380, 548681709, 548681780, 548682466, 548683016, 548683105, 548683107, 548683109, 548683111, 548683113, 548683349, 548683480, 548683481, 548683903, 548684047, 548684080, 548684125, 548684129, 548684131, 548684150, 548684155, 548684208, 548684468, 548684470, 548684471, 548684472, 548684473, 548684488, 548684543, 548684614, 548684680, 548684735, 548684743, 548684909, 548684951, 548684990, 548685101, 548685165, 548685932, 548685933, 548686271, 549770012, 549770013, 549770016, 549770017, 549770100, 549770101, 549770242, 549770243, 550451122, 550451179, 550451189, 550451199, 550451200, 550451201, 550451202, 550451204, 550451226, 550451227, 550451258, 550451345, 550451373, 550451843, 550451848, 550451918, 550452004, 550452067, 550452446, 550452672, 550452756, 550452953, 550452965, 550452966, 550452992, 550452993, 550453016, 550453156, 550453194, 550453260, 550453355, 550453681, 550453684, 550453846, 550453918, 550453919, 550453920, 550453921, 550453928, 550453934, 550810031, 550810032, 550810035, 550810036, 550810130, 550810131, 550810171, 550810172, 550810415, 550810417, 550840001, 550840003, 550840005, 550840006, 550840007, 550840008, 550840009, 550840010, 550840011, 550840012, 550840014, 550840023, 550840025, 550840061, 550840062, 550840064, 550840066, 550840071, 551500111, 551500112, 551500113, 551500114, 551500115, 551500116, 551500117, 551500118, 551500119, 551500120, 551500121, 551500122, 551500123, 551500124, 551500127, 551500128, 551500129, 551750029, 551750030, 551750078, 551750086, 551750415, 551750416, 551750638, 551750639, 551750669, 551750700, 551750960, 551750967, 551751203, 551751217, 551751219, 551751220, 551751221, 551751222, 551751223, 551751745, 551751850, 551752319, 551752673, 551752704, 551752836, 551752838, 551752840, 551752841, 551756020, 551756021, 552890019, 552890020, 552890023, 552890024, 552890071, 552890094, 552890182, 552890206, 552890207, 552890240, 552890242, 552890296, 552890370, 552890398, 552890512, 552890517, 552890592, 552890612, 552890707, 552890727, 552890767, 552890845, 553700880, 553700881, 553700882, 553700883, 553700884, 553700885, 553700886, 553700887, 553700891, 553700892, 557000007, 557000044, 557000075, 557000081, 557000106, 557000166, 557000181, 557000207, 557000230, 557000264, 557000306, 557000308, 557000332, 557000374, 557000375, 557000404, 557000406, 557000416, 557000418, 557000440, 557000444, 557000456, 557000467, 557000468, 557000471, 557000538, 557000560, 557000572, 557000653, 557000687, 558290606, 558290607, 558290610, 558290611, 558290625, 558290626, 558290635, 558290636, 558290689, 558290690, 558870032, 558870139, 558870215, 558870216, 558870218, 558870393, 558870463, 558870622, 558870629, 558870637, 558870659, 558870691, 558870704, 558870714, 558870733, 558870790, 558870814, 558870817, 558870823, 558870830, 558870862, 558870867, 558870930, 558870980, 558870982, 558870993, 559530130, 559530149, 559530671, 559530673, 559530716, 559530724, 559530747, 559530751, 572370001, 572370028, 572370029, 572370030, 572370031, 572370032, 572370033, 572370040, 572370041, 574800455, 574800456, 575060201, 575060202, 575060208, 577836038, 578660106, 578663129, 578663613, 578663614, 578666050, 578666051, 578667218, 580160103, 580160104, 580160105, 580160106, 580160107, 580160108, 580160121, 580160122, 580160146, 580160147, 580160148, 580160149, 580160180, 580160356, 580160512, 580160643, 580160661, 580161000, 580161001, 580161002, 580161003, 580161004, 580161005, 580161006, 580161007, 580161008, 580161009, 580161011, 580161012, 580161026, 580161027, 580161028, 580161029, 580161031, 580161032, 580161033, 580161034, 580161054, 580161062, 580161780, 580161837, 580164138, 580164139, 580164808, 580164842, 580164869, 580164990, 584630002, 584690001, 584690007, 584690008, 584690010, 584690016, 584690070, 584690080, 584690113, 584690114, 584690115, 584690116, 584690118, 584690119, 584690252, 584690253, 584691130, 584691140, 584691150, 584691160, 584691180, 584691190, 584692520, 584692537, 588640028, 588640029, 588640033, 588640149, 588640150, 588640151, 588640379, 588640611, 588640612, 588640663, 588640675, 588640688, 588640697, 588640740, 588640767, 588640777, 589180111, 589180112, 596300142, 597621020, 597621021, 597621022, 597621023, 597621050, 597621534, 597621537, 597720035, 597720036, 597726028, 597726048, 597726058, 599115900, 599115901, 599115902, 599301573, 599301611, 603460013, 603460049, 603460050, 603460066, 603460068, 603460069, 603460070, 603460071, 603460072, 603460074, 603460082, 603460089, 603460091, 603460095, 603460100, 603460113, 603460150, 603460194, 603460221, 603460229, 603460243, 603460244, 603460293, 603460345, 603460364, 603460414, 603460450, 603460480, 603460485, 603460546, 603460561, 603460577, 603460590, 603460593, 603460634, 603460655, 604290021, 604290022, 604290023, 604290024, 604290059, 604290147, 604290148, 604290238, 604320065, 604320070, 605050686, 605050687, 605050688, 605050773, 605052539, 605052540, 605052541, 607930130, 607930131, 607930600, 607930601, 607930602, 607930700, 607930701, 607930702, 614230800, 614230805, 614230810, 614230815, 614230820, 614230825, 614230830, 614230835, 614230840, 614230845, 615700085, 615700086, 615700139, 615700140, 615700141, 615700142, 615700143, 615700144, 615700146, 615700147, 615700148, 615700149, 615700150, 619190015, 619190016, 619190017, 619190020, 619190041, 619190135, 619190202, 619190355, 619190366, 619190393, 619190399, 619190401, 619190424, 619190430, 619190521, 619190633, 619190637, 619190649, 619190665, 619190672, 619190693, 619190716, 625840237, 625840238, 631870014, 631870044, 631870045, 631870200, 631870327, 631870399, 631870423, 631870488, 631870756, 631870803, 631870826, 631870880, 631870926, 633040176, 633040509, 633040514, 633040515, 633040565, 633040567, 633040654, 633040655, 633040713, 633040753, 633040754, 633040760, 633040761, 633040762, 633040763, 633040768, 633040969, 633040970, 633040977, 633040979, 633230300, 633230304, 633230309, 633230320, 633230323, 633230324, 633230327, 633230328, 633230330, 633230368, 633230369, 633230370, 633230380, 633230381, 633230387, 633230388, 633230389, 633230390, 633230391, 633230399, 633230404, 633230704, 633230705, 633230707, 633230708, 636264263, 636291236, 636291248, 636291257, 636291350, 636291351, 636291614, 636291615, 636291759, 636292609, 636292610, 636292738, 636292871, 636292895, 636294216, 636294217, 636294218, 636294226, 636294228, 636294229, 636294236, 636294237, 636294239, 636294256, 636294263, 636294264, 636294265, 636294266, 638740101, 638740102, 638740103, 638740108, 638740113, 638740114, 638740115, 638740116, 638740117, 638740123, 638740143, 638740144, 638740145, 638740146, 638740147, 638740148, 638740170, 638740232, 638740233, 638740239, 638740240, 638740249, 638741112, 638741226, 646790012, 646790034, 646790056, 646790679, 646790698, 646790699, 646790700, 647640702, 658620014, 658620015, 658620016, 658620017, 658620070, 658620071, 658620175, 658620176, 658620501, 658620502, 658620503, 658620533, 658620534, 658620535, 658620706, 658620707, 661160152, 661160157, 661160167, 661160168, 661160240, 661160244, 661160249, 661160262, 661160272, 661160289, 662670021, 662670022, 662670023, 662670024, 662670025, 662670072, 662670073, 662670159, 662670160, 662670444, 662670712, 662670764, 662670857, 662670859, 662670991, 662670992, 662670993, 662670994, 662671001, 662671002, 662671301, 662671309, 662883200, 663360074, 663360082, 663360095, 663360113, 663360229, 663360293, 663360364, 663360368, 663360414, 663360480, 663360593, 663360634, 663360648, 663360655, 666851001, 666851002, 666851011, 666851012, 668600011, 668600012, 668600013, 668600015, 669930418, 670460020, 670460575, 670460576, 672530140, 672530141, 672530142, 672530143, 672530144, 672530145, 672530146, 672530147, 672530148, 672530149, 672530150, 672530180, 672530181, 672530182, 672530183, 672530200, 672530201, 672530202, 672530203, 674570226, 674570230, 674570348, 674570349, 674570350, 674570351, 674570352, 674570353, 674570404, 674570521, 674570522, 674570523, 674570649, 680710063, 680711526, 680711545, 680711546, 680840235, 680840236, 681150025, 681150026, 681150027, 681150028, 681150029, 681150030, 681150032, 681150033, 681150104, 681150105, 681150268, 681150269, 681150386, 681150463, 681150488, 681150582, 681150904, 681150990, 681150991, 681150993, 681150994, 682581100, 682581968, 682581969, 682581997, 682581999, 682583026, 682583052, 682588909, 682588910, 682588911, 682588914, 683870428, 683870430, 683870580, 683870585, 684530142, 687887139, 687887141, 687887155, 687887160, 694420030, 695430103, 695430104, 706550099, 706550109, 708600112, 708600113, 708600114, 708600115, 708600116, 708600117, 708600118, 708600119, 708820111, 708820112, 708820113, 708820114, 709340033, 709340035, 709340038, 709340039, 709340053, 709340054, 709340057, 709340064, 709340086, 709340087, 709340110, 709340126, 709340140, 709340147, 709340148, 709340154, 709340156, 709340162, 712050014, 712050035, 764390103, 764390104, 999990000, 999990001, 999990002, 999990003, 999990005, 999990007, 999990023, 999990055, 999990097, 999990220, 999990222, 999990357, 999991112, 999993265, 999995268, 999996000, 999996565, 999998887 | Oral Penicillin |
| Ciprofloxacin (based on 9-digit substring) | NDC | 003781745, 103700108, 551110423, 620370938, 000851778, 504190789, 548685044, 004084765, 125278897, 003781743, 103700107, 234907664, 545695899, 551110422, 620370942, 000851775, 504190788, 529590855, 545695510, 548684734, 139130001, 154560001, 663360994, 125278889, 617860264, 002471524, 333580080, 551110125, 685330013, 000260511, 550452621, 055111125, 005550380, 681800393, 000268553, 000851773, 125278553, 230708553, 504190773, 000268897, 125378897, 050419788, 062037942, 000268512, 000930863, 001432036, 001439927, 001725311, 001822631, 001850442, 002470197, 002472044, 003781322, 003787097, 004406290, 004407290, 004800863, 005271384, 005550694, 005550814, 007811763, 009045842, 101350474, 105440147, 105440603, 105440604, 105440673, 126340825, 131070076, 134110450, 138631003, 162520514, 165710411, 165900371, 167140651, 216950411, 234905322, 242360383, 242360582, 332610399, 353560803, 353560928, 422910219, 430630410, 458650440, 493490035, 493490246, 493490431, 493490662, 498561308, 498840637, 499990333, 500900867, 500900868, 500901685, 500902146, 500903160, 500903573, 504363135, 510790181, 510790218, 510790402, 511292853, 511294060, 511294167, 516550115, 516550525, 516724085, 521250245, 525494085, 525550769, 525550774, 529590739, 530020279, 530022790, 539783312, 543480662, 545695584, 545698318, 548684898, 549070709, 550453142, 550480069, 551110126, 551542086, 551543496, 551545852, 551546177, 552890823, 557000279, 557000744, 558870904, 578663135, 580160137, 581180411, 588640806, 591150030, 604290742, 605051308, 607600512, 614420222, 617860097, 619190371, 625405311, 631260251, 631870142, 633040709, 636291009, 636291326, 636296358, 636297648, 637390427, 637390700, 642050927, 650150108, 654130769, 658620076, 663360433, 672280032, 672280286, 672960176, 672960237, 672960261, 672960608, 672960620, 672961097, 672961575, 675440796, 676680121, 677670137, 680710050, 680710055, 680711893, 680713013, 680714260, 680714387, 680714813, 680840069, 681150464, 685330014, 687880709, 687889006, 687889280, 687889724, 691170008, 705180464, 705181090, 705181975, 707860411, 709340068, 713350408, 713350836, 000260512, 000851758, 001791224, 004033027, 125278512, 126340423, 504190758, 516550537, 529590171, 545691648, 548680990, 550451469, 551544806, 551751895, 552890459, 558870792, 578666250, 580160116, 588640775, 603460433, 604910134, 625840335, 645790017, 661050108, 662670431, 681150081, 010544603, 016571411, 043063410, 049349662, 010544604, 013107076, 016252514, 035356803, 035356928, 051079181, 051079402, 052125245, 055111126, 055289823, 061442222, 063304709, 010370107, 010370108, 062037938, 050419789, 005550379, 681800392, 000268551, 000851777, 125278551, 230708551, 504190777, 545695824, 000268889, 553700799, 000268513, 000930864, 001432037, 001439928, 001725312, 001790054, 001790188, 001791510, 001791982, 001822721, 001850451, 002470167, 002472042, 003781323, 003787098, 004406291, 004407291, 004800864, 005271385, 005550695, 005550815, 007811765, 009045843, 009046378, 101350475, 105440036, 105440079, 105440148, 105440307, 105440432, 105440605, 105440606, 105440607, 105440608, 105440884, 105440943, 118190323, 118190351, 118190360, 118190363, 126340769, 131070077, 134110314, 138631004, 162520515, 165710412, 165900054, 167140652, 188370245, 216950210, 234900320, 234905323, 242360030, 242360648, 246580185, 246580250, 332610164, 333580081, 353560732, 353560733, 353560846, 412840515, 425490507, 425490598, 427080002, 427080088, 428520001, 430630053, 430630427, 430630547, 430630869, 430630923, 433530472, 433530473, 433530924, 436830115, 447950001, 458650471, 474630068, 493490036, 493490086, 493490194, 493490291, 493490453, 493490612, 493490634, 493490854, 498561309, 498840638, 499990334, 500900863, 500900864, 500901648, 500901649, 500902265, 500903130, 500903791, 504360077, 504360412, 504366041, 510790182, 510790233, 510790403, 511292838, 511292859, 511292889, 516550118, 516724086, 521250069, 523430067, 525494086, 525550770, 525550775, 529590730, 530020264, 530022640, 532170186, 534010004, 534010005, 534010301, 538080074, 538080223, 538080306, 538080349, 538081073, 539783075, 545695574, 545698311, 548684858, 549070710, 550453080, 550453467, 550480068, 551110127, 551542081, 551545376, 551545851, 551546164, 552890821, 555670098, 557000481, 557000608, 558870584, 578666041, 580160953, 581180127, 588640833, 591150029, 604290042, 604290743, 605051309, 607600518, 607600815, 614420223, 619190054, 619190361, 620340016, 631260252, 631870017, 631870250, 633040710, 636291010, 636291724, 636295154, 636296360, 636297717, 636297786, 637390400, 637390559, 638741086, 642050928, 647250387, 650150109, 654130770, 658620077, 661160480, 661160508, 662670716, 662670919, 663360525, 663360527, 663360903, 672280033, 672280235, 672280376, 672280452, 672960075, 672960118, 672960210, 672960543, 672960579, 672960580, 672960599, 672960853, 672961318, 675440797, 675440823, 675440867, 676680122, 677670138, 680710229, 680714122, 680714201, 680714492, 680714576, 680714627, 680714849, 680840070, 681150446, 682589237, 683870535, 685330015, 686450030, 686450060, 687880127, 687880710, 687881385, 687889012, 687889024, 687889228, 687889330, 691170009, 705180010, 705180368, 705180693, 705181278, 707860515, 709340045, 709340093, 713350070, 716100081, 716100109, 716100159, 759210412, 764130164, 000260513, 000851754, 001791225, 004030601, 125278513, 126340689, 499990061, 504190754, 516550385, 529590036, 545691723, 545694820, 548680939, 550451494, 551544801, 551751896, 552890371, 558870858, 578666251, 580160117, 588640637, 603460031, 604910139, 607600513, 618070035, 625840336, 638740107, 645790018, 661050109, 662670058, 672960028, 681150082, 511293524, 010544079, 010544608, 016571412, 016590054, 035356846, 075921412, 053401004, 010544605, 010544607, 011819363, 013107077, 016252515, 024658250, 043063053, 043063427, 049999334, 051079182, 051079403, 052125069, 055111127, 055289821, 061442223, 063304710, 068645060, 000930865, 001432038, 001439929, 001725313, 001822722, 001850470, 002470928, 003781324, 003787099, 004407292, 004800865, 005271386, 005550696, 005550816, 007811767, 101350476, 138631005, 162520516, 165710413, 165900735, 167140653, 234905324, 332610367, 353560264, 430630767, 458650530, 498561310, 498840639, 510790234, 511292860, 516724087, 525494087, 525550771, 525550776, 529590734, 545695583, 548685023, 550453081, 551110128, 551545853, 551546187, 552890826, 558870292, 580160957, 581180413, 591150028, 604290744, 605051310, 614420224, 619190735, 631260253, 631870990, 633040711, 636293868, 650150110, 654130771, 658620078, 663360692, 672961269, 672961389, 676680123, 677670139, 680710331, 680840071, 683870534, 685330016, 685330516, 713350339, 713351024, 000260514, 000268514, 000851756, 001791226, 004033511, 125278514, 529590037, 530020268, 545692488, 548681184, 550451650, 551544807, 551751897, 552890717, 578667132, 580160118, 604910140, 645790019, 016571413, 016252516, 016590735, 055111128, 055289826, 061442224, 063304711, 474631004, 682581079, 515521229, 387790534, 519273634, 629912518, 055111422, 049349035, 049349246, 049349431, 021695411, 049999333, 050419758, 016714651, 052959739, 060429742, 065862076, 068084069, 016590421, 021695259, 061314656, 016571120, 024208444, 052125483, 052125537, 055111423, 010544606, 042852001, 049349036, 049349086, 049349291, 049349453, 049349634, 049349854, 066116508, 053401005, 050419754, 049349194, 049349612, 011819323, 011819351, 011819360, 016714652, 021695210, 035356732, 052959730, 060429743, 063739559, 065862077, 066116480, 068084070, 013913001, 016714653, 052959734, 065862078, 068084071, 016590053, 050419773, 050419777, 033261164, 033261367, 033261399, 060760512, 719910003, 621570419 | Oral ciprofloxacin |
|  |  |  |  |
|  |  |  |  |
|  |  |  |  |
|  |  |  |  |
|  |  |  |  |
|  |  |  |  |
|  |  |  |  |
|  |  |  |  |
|  |  |  |  |
|  |  |  |  |
|  |  |  |  |
|  |  |  |  |
|  |  |  |  |
|  |  |  |  |
|  |  |  |  |
|  |  |  |  |
|  |  |  |  |
|  |  |  |  |
|  |  |  |  |
|  |  |  |  |
|  |  |  |  |
|  |  |  |  |
|  |  |  |  |
|  |  |  |  |
|  |  |  |  |
|  |  |  |  |
|  |  |  |  |
|  |  |  |  |
|  |  |  |  |
|  |  |  |  |
|  |  |  |  |
|  |  |  |  |
|  |  |  |  |
|  |  |  |  |
|  |  |  |  |
|  |  |  |  |
|  |  |  |  |
|  |  |  |  |
|  |  |  |  |
|  |  |  |  |
|  |  |  |  |
|  |  |  |  |
|  |  |  |  |
|  |  |  |  |
|  |  |  |  |
|  |  |  |  |
|  |  |  |  |
|  |  |  |  |
|  |  |  |  |
|  |  |  |  |
|  |  |  |  |
|  |  |  |  |
|  |  |  |  |
|  |  |  |  |
|  |  |  |  |
|  |  |  |  |
|  |  |  |  |
|  |  |  |  |
|  |  |  |  |
|  |  |  |  |
|  |  |  |  |
