# Supplementary material for: Characteristics of and meningococcal disease prevention strategies for commercially insured persons receiving eculizumab in the United States
Source: PLoS One. 2020 Nov 12;15(11):e0241989. doi: 10.1371/journal.pone.0241989 (PMC7660549; doi:10.1371/journal.pone.0241989)
Supplement: S2 Table — (DOCX) [file pone.0241989.s002.docx]

S2 Table. International Classification of Diseases (ICD) 9/10 codes used to define medical conditions and their description.

| Medical conditions | ICD-9 Code | Description of code | ICD-10 Code | Description of code |
| --- | --- | --- | --- | --- |
| PNH | 283.2 | Hemoglobinuria due to hemolysis from external causes | D59.5 | Paroxysmal nocturnal hemoglobinuria |
| aHUS | 283.11 | Hemolytic uremic syndrome | D59.3 | Hemolytic uremic syndrome |
| Typical HUS | 283.11 and either  041.4* or  004.0 | Hemolytic uremic syndrome and either  Shiga toxin-producing *Escherichia coli* with O157, non-O157 (with known O group), or unspecified O group, non-Shiga toxin-producing *E. coli*, or  *Shigella dysenteriae* | D59.3 and either  B96.2,  J13, or  A03.9 | Hemolytic uremic syndrome and either E. coli as the cause of diseases classified elsewhere,  pneumonia due to *Streptococcus pneumoniae*, or  shigellosis (unspecified) |
| gMG | 358.00 | Myasthenia gravis without (acute) exacerbation | G70.00 | Myasthenia gravis without (acute) exacerbation |
| Transplant | V42.0 | Kidney transplant | Z94.0 | Kidney transplant |
|  | V42.1 | Heart transplant | Z94.1 | Heart transplant |
|  | V42.2 | Heart valve transplant | Z94.3 | Heart and lungs transplant |
|  | V42.3 | Skin transplant | Z94.5 | Skin transplant |
|  | V42.4 | Bone transplant | Z94.6 | Bone transplant |
|  | V42.5 | Cornea transplant | Z94.7 | Cornea transplant |
|  | V42.6 | Lung transplant | Z94.2 | Lung transplant |
|  | V42.7 | Liver transplant | Z94.4 | Liver transplant |
|  | V42.81 | Bone marrow transplant | Z94.81 | Bone marrow transplant |
|  | V42.82 | Peripheral stem cell transplant | Z94.84 | Stem cell transplant |
|  | V42.83 | Pancreas transplant | Z94.83 | Pancreas transplant |
|  | V42.84 | Intestine transplant | Z94.82 | Intestine transplant |
|  | V42.89 | Tissue or organ transplant (with site and type NEC) | Z94.89 | Other transplanted organ and tissue |
|  | V42.9 | Tissue or organ transplant | Z94.9 | Tissue or organ transplant |
| Other causes of thrombotic microangiopathy | 286.6 | Disseminated intravascular coagulation [defibrination syndrome] | D65 | Disseminated intravascular coagulation [defibrination syndrome] |
|  | 642.5 | Severe pre-eclampsia | O14.2 | HELLP (hemolysis, elevated liver enzymes and low platelet count) |
|  | 710.1 | Systemic sclerosis | M34.0 | Progressive systemic sclerosis |
|  |  |  | M34.9 | Systemic sclerosis (unspecified) |
|  | 289.81 | Primary hypercoagulable state | D68.61 | Antiphospholipid syndrome |
|  | 710.0 | Systemic lupus erythematosus | M32.10 | Systemic lupus erythematosus |
| Aplastic anemia | 284.01 | Constitutional red blood cell aplasia | D61.* | Constitutional red blood cell aplasia, other constitutional aplastic anemia, drug-induced aplastic anemia, aplastic anemia due to other external agents, idiopathic aplastic anemia, antineoplastic chemotherapy induced pancytopenia, other drug-induced pancytopenia, other pancytopenia, myelophthisis, other specified aplastic anemias and other bone marrow failure synrdome, aplastic anemia (unspecified) |
|  | 284.09 | Other constitutional aplastic anemia |  |  |
|  | 284.89 | Other specific aplastic anemia |  |  |
|  | 284.19 | Other pancytopenia |  |  |
|  | 284.9 | Aplastic anemia (unspecified) |  |  |
| Other hemolytic anemias (both acquired and hereditary) | 282.0 | Spherocytosis | D58.* | Hereditary hemolytic anemias (spherocytosis, elliptocytosis, other hemoglobinopathies, other specified hereditary hemolytic anemias, hereditary hemolytic anemia (unspecified) |
|  | 282.1 | Elliptocytosis |  |  |
|  | 282.2 | Anemias due to disorders of glutathione metabolism | D59.6 | Hemoglobinuria due to hemolysis from other external causes |
|  | 282.3 | Other hemolytic anemias due to enzyme deficiency | D59.8 | Other acquired hemolytic anemias |
|  | 282.7 | Other hemoglobinopathies |  |  |
|  | 282.8 | Other specified hereditary hemolytic anemias |  |  |
|  | 282.9 | Hereditary hemolytic anemia (unspecified) |  |  |
|  | 283.0 | Autoimmune hemolytic anemias | D59.0 | Drug-induced autoimmune hemolytic anemia |
|  |  |  | D59.1 | Other autoimmune hemolytic anemias |
|  | 283.19 | Non-autoimmune hemolytic anemia (unspecified) | D59.2 | Drug-induced non-autoimmune hemolytic anemia |
|  | 283.10 | Other non-autoimmune hemolytic anemias | D59.4 | Other non-autoimmune hemolytic anemias |
|  | 283.9 | Acquired hemolytic anemia (unspecified) | D59.9 | Acquired hemolytic anemia (unspecified) |
| Blood cancer | 200-208 | Neoplasms of the lymphatic and hematopoietic tissue | C81-C96 | Malignant neoplasms, stated or presumed to be primary, of lymphoid, haemetapoietic and related tissue |
| All other cancers | 140-149 | Neoplasms of the lip, oral cavity, and pharynx | C00-C14 | Malignant neoplasms of the lip, oral cavity, and pharynx |
|  | 150-159 | Neoplasms of the digestive organs and peritoneum | C15-C26 | Malignant neoplasms of digestive organs |
|  | 160-165 | Neoplasms of the respiratory and intrathoracic organs | C30-C39 | Malignant neoplasms of the respiratory system and intrathoracic organs |
|  | 170-175 | Neoplasms of the bone, connective tissue, skin, and breast | C40-C41 | Malignant neoplasms of the bone and articular cartilage |
|  | 176 | Kaposi's sarcoma | C43-C44 | Malignant neoplasms of the skin |
|  |  |  | C45-C49 | Malignant neoplasms of the connective and soft tissue |
|  | 179-189 | Neoplasms of the genitourinary organs | C50-C58 | Malignant neoplasms of the breast and female genital organs |
|  |  |  | C60-C63 | Malignant neoplasms of the male genital organs |
|  |  |  | C64-C68 | Malignant neoplasms of the urinary organs |
|  |  |  | C69-C72 | Malignant neoplasms of the eye, brain, and central nervous system |
|  | 209 | Neuroendocrine tumors | C73-C75 | Malignant neoplasms of the endocrine glands and related structures |
|  |  |  | C76-C80 | Malignant neoplasms, secondary and ill-defined |
|  | 190-199 | Neoplasms of the other and unspecified sites | C97 | Malignant neoplasms of independent (primary) multiple sites |
|  | 230-234 | Carcinoma in situ | D00-D09 | In situ neoplasms |
|  | 210-229 | Benign neoplasms | D10-D36 | Benign neoplasms |
|  | 235-238 | Neoplasms of uncertain behavior | D37-D48 | Neoplasms of uncertain or unknown behavior |
|  | 239 | Neoplasms of unspecified nature |  |  |
| Other kidney diagnoses (excluding kidney transplant) | 580 | Acute glomerulonephritis | N00 | Acute nephritic syndrome |
|  |  |  | N01 | Rapidly progressive nephritis syndrome |
|  |  |  | N02 | Recurrent and persistent hematuria |
|  | 582 | Chronic glomerulonephritis | N03 | Chronic nephritic syndrome |
|  | 581 | Nephrotic syndrome | N04 | Nephrotic syndrome |
|  | 583 | Nephritis and nephropathy | N05 | Unspecified nephritic syndrome |
|  |  |  | N06 | Isolated proteinuria with specified morphological lesion |
|  |  |  | N07 | Hereditary nephropathy, not elsewhere classified |
|  |  |  | N08 | Glomerular disorders in diseases classified elsewhere |
|  | 590 | Infections of kidney | N10 | Acute tubulo-interstitial nephritis |
|  |  |  | N11 | Chronic tubulo-interstitial nephritis |
|  |  |  | N12 | Tubulo-interstitial nephritis not specified as acute or chronic |
|  | 591 | Hydronephrosis | N13 | Obstructive and reflux uropathy |
|  |  |  | N14 | Drug- and heavy-metal-induced tubulo-interstitial and tubular conditions |
|  |  |  | N15 | Other renal tubulo-interstitial diseases |
|  |  |  | N16 | Renal tubulo-interstitial disorders in diseases classified elsewhere |
|  | 584 | Acute renal failure | N17 | Acute kidney failure |
|  | 585 | Chronic renal failure | N18 | Chronic kidney disease |
|  | 586 | Renal failure (unspecified) | N19 | Unspecified kidney failure |
|  | 592 | Calculus of kidney and ureter | N20 | Calculus of kidney and ureter |
|  | 594 | Calculus of lower urinary tract | N21 | Calculus of lower urinary tract |
|  |  |  | N22 | Calculus of urinary tract in diseases classified elsewhere |
|  |  |  | N23 | Unspecified renal colic |
|  | 588 | Disorders resulting from impaired renal function | N25 | Disorders resulting from impaired renal tubular funciton |
|  | 587 | Renal sclerosis (unspecified) | N26 | Unspecified contracted kidney |
|  | 589 | Small kidney or unknown cause | N27 | Small kidney of unknown cause |
|  | 593 | Other disorders of kidney and ureter | N28 | Other disorders of kidney and ureter not elsewhere classified |
|  |  |  | N29 | Other disorders of kidney and ureter in disease classified elsewhere |
| Joint diagnoses | 711 | Arthropathy associated with infections | M00 | Pyogenic arthritis |
|  |  |  | M01 | Direct infections of joint in infectious and parasitic diseases classified elsewhere |
|  |  |  | M02 | Reactive arthropathies |
|  |  |  | M03 | Postinfective and reactive arthropathies in diseases classified elsewhere |
|  |  |  | M05 | Seropositive rheumatoid arthritis |
|  | 714 | Rheumatoid arthritis and other inflammatory polyarthropathies | M06 | Other rheumatoid arthritis |
|  |  |  | M07 | Psoriatic and enteropathic arthropathies |
|  |  |  | M08 | Juvenile arthritis |
|  |  |  | M09 | Juvenile arthritis in diseases classified elsewhere |
|  |  |  | M10 | Gout |
|  | 712 | Crystal arthropathies | M11 | Other crystal arthropathies |
|  | 716 | Other and unspecified arthropathies | M12 | Other specific arthropathies |
|  |  |  | M13 | Other arthritis |
|  | 713 | Arthropathy endrocrine disorders | M14 | Arthropathies in other diseases classified elsewhere |
|  | 715 | Osteoarthrosis and allied disorders | M15 | Polyarthrosis |
|  |  |  | M16 | Coxarthrosis |
|  |  |  | M17 | Gonarthrosis |
|  |  |  | M18 | Arthrosis of first carpometacarpal joint |
|  |  |  | M19 | Other arthrosis |
|  |  |  | M20 | Acquired deformities of fingers and toes |
|  |  |  | M21 | Other acquired deformities of limbs |
|  |  |  | M22 | Disorders of patella |
|  | 717 | Internal derangement of knee | M23 | Internal derangement of knee |
|  | 718 | Other derangement of joint | M24 | Other specific joint derangements |
|  | 718 | Other and unspecified disorders of joint | M25 | Other joint disorders not elsewhere classified |
|  |  |  | S03 | Dislocation, sprain and strain of joints and ligaments of head |
|  |  |  | S13 | Dislocation, sprain and strain of joints and ligaments at neck level |
|  |  |  | S23 | Dislocation, sprain and strain of joints and ligaments of thorax |
|  | 846 | Sprains and strains of sacroiliac region | S33 | Dislocation, sprain and strain of joints and ligaments of lumbar spine and pelvis |
|  | 840 | Sprains and strains of shoulder and upper arm | S43 | Dislocation, sprain and strain of joints and ligaments of shoulder girdle |
|  | 841 | Sprains and strains of elbow and forearm | S53 | Dislocation, sprain and strain of joints and ligaments of elbow |
|  | 842 | Sprains and strains of wrist and hand | S63 | Dislocation, sprain and strain of joints and ligaments at wrist and hand level |
|  | 843 | Sprains and strains of hip and thigh | S73 | Dislocation, sprain and strain of joint and ligaments of hip |
|  | 844 | Sprains and strains of knee and leg | S83 | Dislocation, sprain and strain of joints and ligaments of knee |
|  | 845 | Sprains and strains of ankle and foot | S93 | Dislocation, sprain and strain of joints and ligaments at ankle and foot level |
|  | 847 | Sprains and strains of other and unspecified parts of back |  |  |
|  | 848 | Other and ill-defined sprains and strains |  |  |
| Meningococcal disease | 036.0 | Meningococcal meningitis | A39.0 | Meningococcal meningitis |
|  | 036.1 | Meningococcal encephalitis | A39.81 | Meningococcal encephalitis |
|  | 036.2 | Meningococcemia | A39.2 | Acute meningococcemia |
|  |  |  | A39.3 | Chronic meningococcemia |
|  |  |  | A39.4 | Meningococcemia, unspecified |
|  | 036.3 | Waterhouse-Friderichsen syndrome, meningococcal | A39.1 | Waterhouse-Friderichsen syndrome |
|  | 036.40 | Meningococcal carditis | A39.50 | Meningococcal carditis, unspecified |
|  | 036.41 | Meningococcal pericarditis | A39.53 | Meningococcal pericarditis |
|  | 036.42 | Meningococcal endocarditis | A39.51 | Meningococcal endocarditis |
|  | 036.43 | Meningococcal myocarditis | A39.52 | Meningococcal myocarditis |
|  | 036.81 | Meningococcal optic neuritis | A39.82 | Meningococcal retrobulbar neuritis |
|  | 036.82 | Meningococcal arthropathy | A39.83 | Meningococcal arthritis |
|  |  |  | A39.84 | Postmeningococcal arthritis |
|  | 036.89 | Other meningococcal infections | A39.89 | Other meningococcal infections |
|  | 036.9 | Meningococcal infection, unspecified | A39.9 | Meningococcal infection, unspecified |
| Gonococcal disease | 098.0 | Gonococcal infection (acute) of lower genitourinary tract, unspecified | A54.00 | Gonococcal infection of lower genitourinary tract, unspecified |
|  | 098.10 | Gonococcal infection (acute) of upper genitourinary tract, site unspecified | A54.29 | Other gonococcal genitourinary infections |
|  | 098.11 | Gonococcal cystitis (acute) | A54.01 | Gonococcal cystitis and urethritis, unspecified |
|  | 098.12 | Gonococcal prostatitis (acute) | A54.22 | Gonococcal prostatitis |
|  | 098.13 | Gonococcal epididymo-orchitis | A54.23 | Gonococcal infection of other male genital organs |
|  | 098.14 | Gonococcal seminal vesiculitis (acute) | A54.23 | Gonococcal infection of other male genital organs |
|  | 098.15 | Gonococcal cervicitis (acute) | A54.03 | Gonococcal cervicitis, unspecified |
|  | 098.16 | Gonococcal endometritis (acute) | A54.24 | Gonococcal female pelvic inflammatory disease |
|  | 098.17 | Gonococcal salpingitis, specified as acute | A54.29 | Other gonococcal genitourinary infections |
|  | 098.19 | Other gonococcal infection (acute) of upper genitourinary tract | A54.21 | Gonococcal infection of kidney and ureter |
|  | 098.2 | Gonococcal infection, chronic, of lower genitourinary tract | A54.00 | Gonococcal infection of lower genitourinary tract, unspecified |
|  | 098.30 | Chronic gonococcal infection of upper genitourinary tract, site unspecified | A54.29 | Other gonococcal genitourinary infections |
|  | 098.31 | Gonococcal cystitis (chronic) | A54.01 | Gonococcal cystitis and urethritis, unspecified |
|  | 098.32 | Gonococcal prostatitis (chronic) | A54.22 | Gonococcal prostatitis |
|  | 098.33 | Gonococcal epididymo-orchitis (chronic) | A54.23 | Gonococcal infection of other male genital organs |
|  | 098.34 | Gonococcal seminal vesiculitis (chronic) | A54.23 | Gonococcal infection of other male genital organs |
|  | 098.35 | Gonococcal cervicitis (chronic) | A54.03 | Gonococcal cervicitis, unspecified |
|  | 098.36 | Gonococcal endometritis (chronic) | A54.24 | Gonococcal female pelvic inflammatory disease |
|  | 098.37 | Gonococcal salpingitis (chronic) | A54.29 | Other gonococcal genitourinary infections |
|  | 098.39 | Other chronic gonococcal infection of upper genitourinary tract | A54.29 | Other gonococcal genitourinary infections |
|  | 098.40 | Gonococcal conjunctivitis | A54.31 | Gonococcal conjunctivitis |
|  | 098.41 | Gonococcal iridocyclitis | A54.32 | Gonococcal iridocyclitis |
|  | 098.42 | Gonococcal endophthalmia | A54.39 | Other gonococcal eye infection |
|  | 098.43 | Gonococcal keratitis | A54.33 | Gonococcal keratitis |
|  | 098.49 | Other gonococcal infection of eye | A54.30 | Other gonococcal eye infection |
|  | 098.50 | Gonococcal arthritis | A54.42 | Gonococcal arthritis |
|  | 098.51 | Gonococcal synovitis and tenosynovitis | A54.49 | Gonococcal infection of other musculoskeletal tissue |
|  | 098.52 | Gonococcal bursitis | A54.49 | Gonococcal infection of other musculoskeletal tissue |
|  | 098.53 | Gonococcal spondylopathy | A54.41 | Gonococcal spondylopathy |
|  | 098.59 | Other gonococcal infection of joint | A54.40 | Gonococcal infection of musculoskeletal system, unspecified |
|  | 098.6 | Gonococcal pharyngitis | A54.5 | Gonococcal pharyngitis |
|  | 098.7 | Gonococcal infection of anus and rectum | A54.6 | Gonococcal infection of anus and rectum |
|  | 098.81 | Gonococcal keratosis | A54.89 | Other gonococcal infections |
|  | 098.82 | Gonococcal meningitis | A54.81 | Gonococcal meningitis |
|  | 098.83 | Gonococcal pericarditis | A54.83 | Gonococcal heart infection |
|  | 098.84 | Gonococcal endocarditis | A54.83 | Gonococcal heart infection |
|  | 098.85 | Other gonococcal heart disease | A54.83 | Gonococcal heart infection |
|  | 098.86 | Gonococcal peritonitis | A54.85 | Gonococcal peritonitis |
|  | 098.89 | Gonococcal infection of other specified sites | A54.86 | Gonococcal sepsis |
|  |  |  | A54.02 | Gonococcal vulvovaginitis, unspecified |
|  |  |  | A54.09 | Other gonococcal infection of lower genitourinary tract |
|  |  |  | A54.43 | Gonococcal osteomyelitis |
|  |  |  | A54.82 | Gonococcal brain abscess |
|  |  |  | A54.84 | Gonococcal pneumonia |
|  |  |  | A54.89 | Other gonococcal infections |
|  |  |  | A54.9 | Gonococcal infection, unspecified |
| Meningitis not otherwise specified | 322.9 | Meningitis, unspecified | G03.9 | Meningitis, unspecified |
| Sepsis not otherwise specified | 995.91 | Sepsis | A41.9 | Sepsis, unspecified organism |
