# Supplementary material for: Characteristics of and meningococcal disease prevention strategies for commercially insured persons receiving eculizumab in the United States
Source: PLoS One. 2020 Nov 12;15(11):e0241989. doi: 10.1371/journal.pone.0241989 (PMC7660549; doi:10.1371/journal.pone.0241989)
Supplement: S3 Table — (DOCX) [file pone.0241989.s003.docx]

S3 Table. Co-morbidities and potential etiology among eculizumab recipients diagnosed with meningococcal disease, meningitis not otherwise specified and/or sepsis not otherwise specified in the IBM® Marketscan® Commercial Database, 2007–2017.

| **Diagnosis** | **Meningococcal disease** (n=1), N (%) | **Meningitis not otherwise specified (NOS)** (n=3), N (%) | **Sepsis NOS** (n=40), N (%) | **Both meningitis and sepsis NOS** (n=4), N (%) |
| --- | --- | --- | --- | --- |
| Presence of cancer codes within the defined six-week window of first documented eculizumab | 1 (100%) | 1 (33.3%) | 15 (37.5%) | 1 (25.0%) |
| Presence of transplant codes within the defined six-week window of first documented eculizumab | 0 (0%) | 1 (33.3%) | 5 (12.5%) | 0 (0%) |
| Claim source | 1 (100%) inpatient | 3 (100%) inpatient | 31 (77.5%) inpatient 1 (2.5%) ER 8 (20%) not admitted | 4 (100%) inpatient |
| Potential etiology noted in other inpatient claim codes | 1 (100%) meningococcemia | 3 (100%) meningitis NOS only | 2 (5%) *E. coli* 2 (5%) gram-negative bacteria 1 (2.5%) *Klebsiella pneumoniae* and *Enterococcus*  1 (2.5%) methicillin resistant *Staphylococcus aureus* 26 (65%) sepsis NOS only  8 (20%) N/A (not admitted) | 2 (50.0%) meningitis due to bacteria NOS  1 (25.0%) pneumococcal meningitis, pneumococcal sepsis 1 (25.0%) viral meningitis |
