# Supplementary figures and images for: Characteristics of and meningococcal disease prevention strategies for commercially insured persons receiving eculizumab in the United States
Source: PLoS One. 2020 Nov 12;15(11):e0241989. doi: 10.1371/journal.pone.0241989 (PMC7660549; doi:10.1371/journal.pone.0241989)

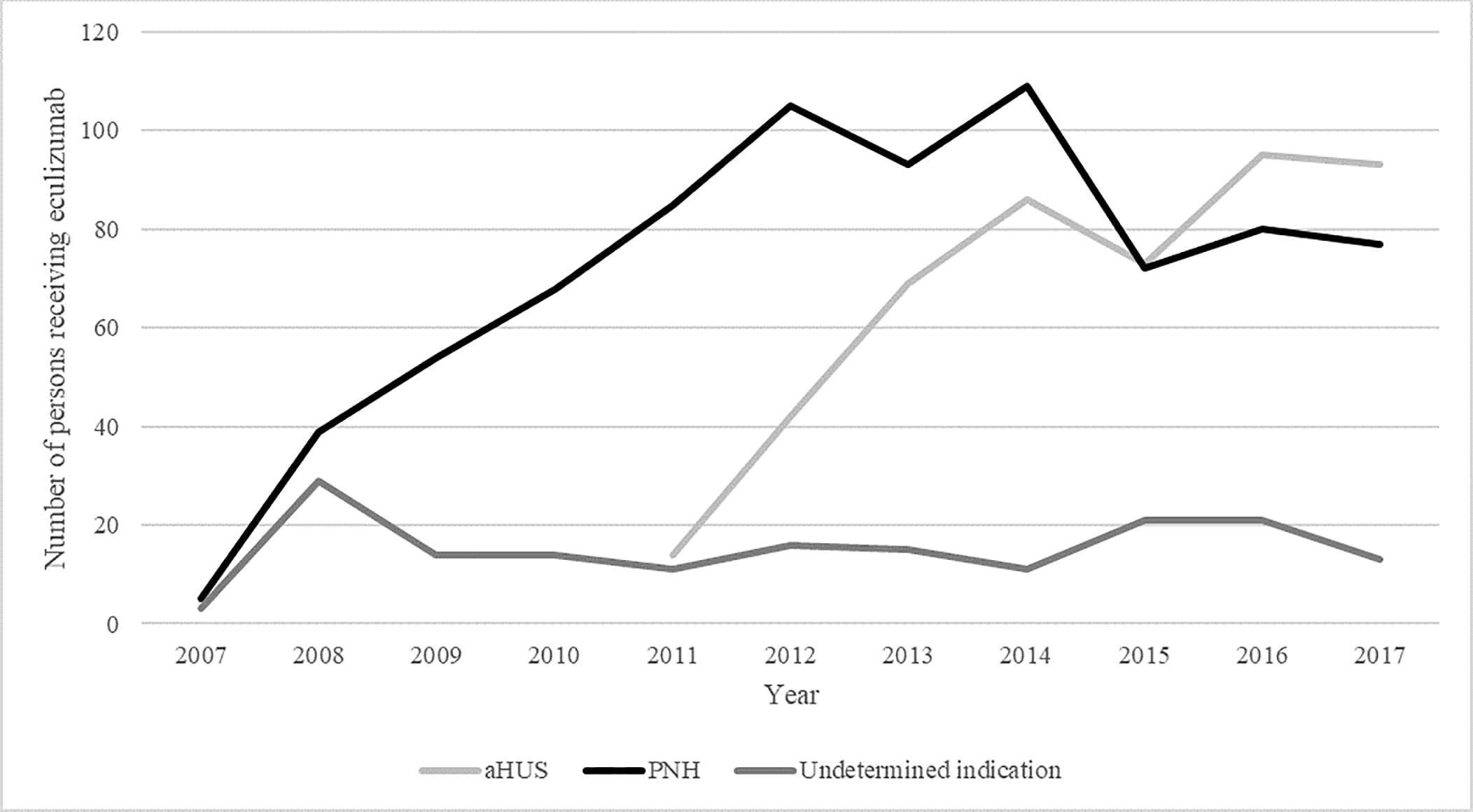

Supplement: S1 Fig — (TIF) [file pone.0241989.s004.tif]

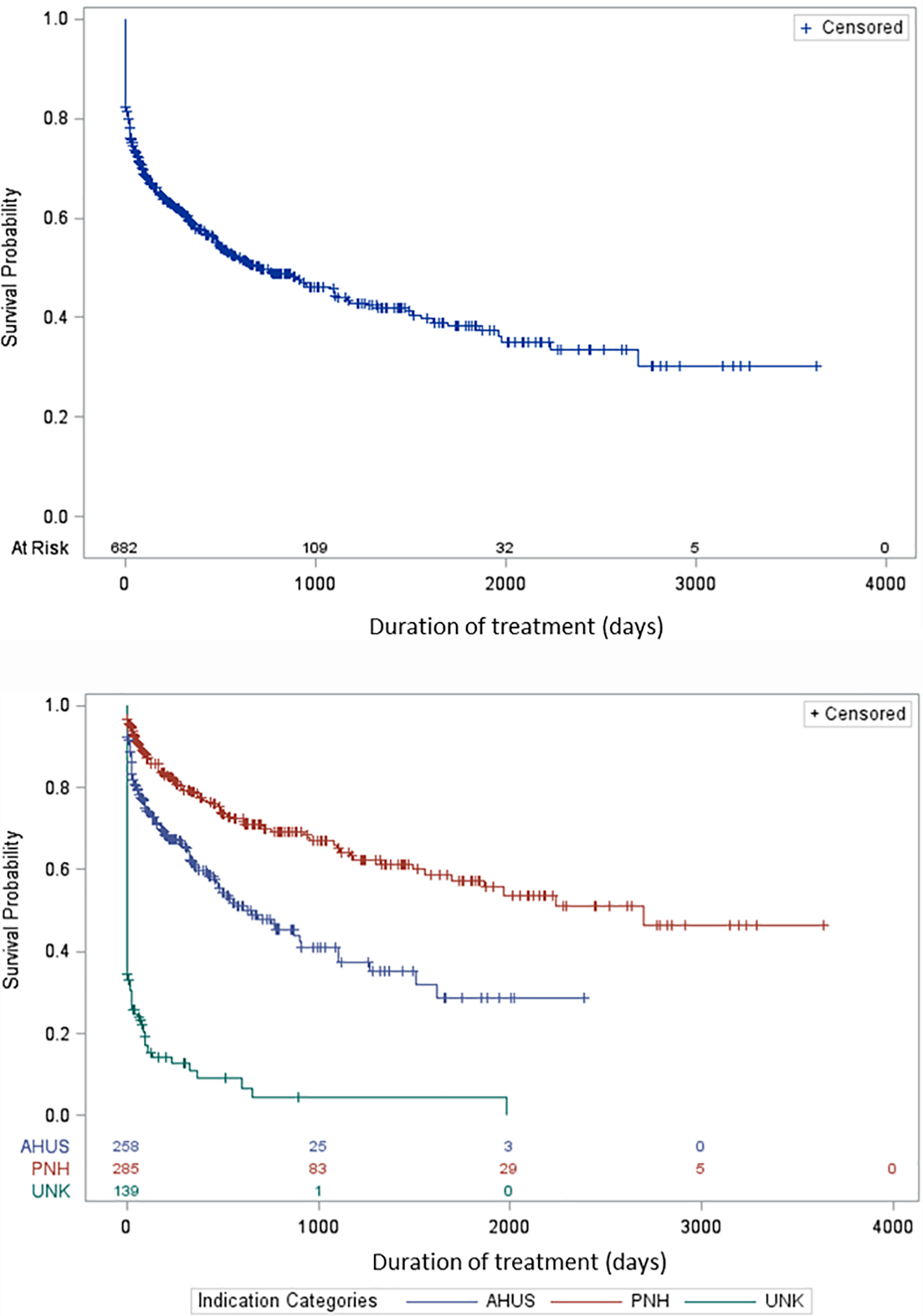

Supplement: S2 Fig — Median duration of eculizumab treatment among (a) all eculizumab recipients in the IBM Marketscan Commercial Database and (b) by indication, 2007–2017. (TIF) [file pone.0241989.s005.tif]

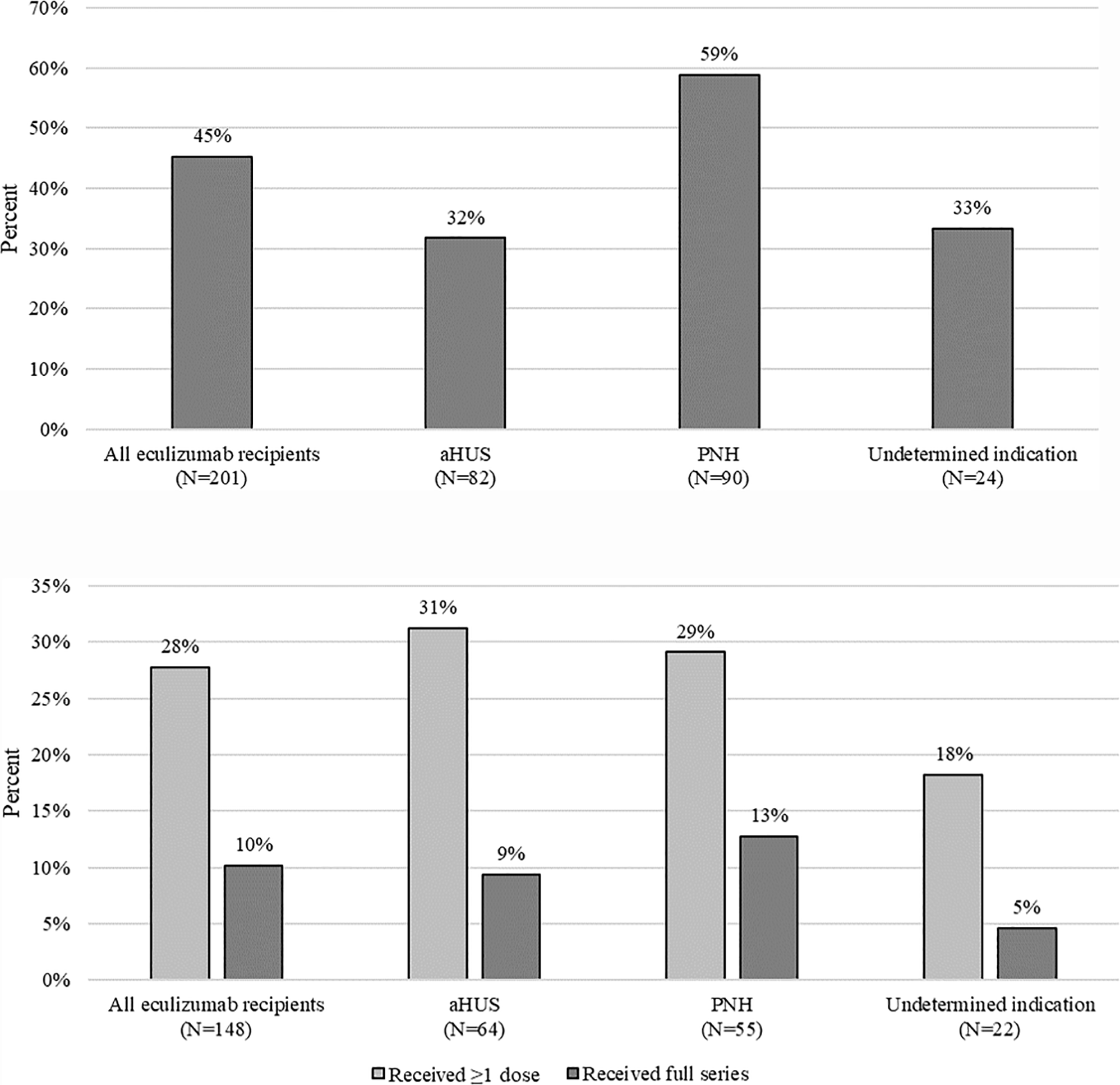

Supplement: S3 Fig — Receipt of a) at least one MenACWY vaccine in the five years prior to the last documented eculizumab dose among eculizumab recipients and b) one or more doses or complete series (2–3 doses) of MenB vaccine, IBM Marketscan Commercial Database, 2007–2017. (TIF) [file pone.0241989.s006.tif]
